# Bifunctional Artificial Enzymes‐Loaded Microgels With LOX‐ and CAT‐Like Activities for Metabolic Reprogramming and Scarless Wound Repair

**DOI:** 10.1002/advs.202521248

**Published:** 2026-03-06

**Authors:** Yongyuan Kang, Pai Peng, Liang Song, Qiaoxuan Wang, Zilong Zhong, Dongchao Qiu, Yunfan Liu, Kefei Zhao, Xiaofei Dong, Changyou Gao

**Affiliations:** ^1^ Zhejiang Key Laboratory of Advanced Organic Materials and Technologies MOE Key Laboratory of Macromolecular Synthesis and Functionalization Department of Polymer Science and Engineering Zhejiang University Hangzhou China; ^2^ Zhejiang Engineering Research Center For Interface Technology of Medical Polymers and Devices Shaoxing Key Laboratory of Healthcare Materials and Application Technology and Center for Healthcare Materials Shaoxing Institute Zhejiang University Shaoxing China; ^3^ Dr. Li Dak Sum & Yip Yio Chin Center for Stem Cell and Regenerative Medicine Zhejiang University Hangzhou China

**Keywords:** artificial enzymes, hypertrophic scars, metabolism reprogramming, microgels, wound healing

## Abstract

Lactate plays a central role in regulating wound metabolism and has been implicated in fibrosis through mechanisms such as histone lactylation and endothelial‐to‐mesenchymal transition. However, strategies to modulate lactate‐driven metabolic imbalance and oxidative stress remain limited. Herein, a metabolism‐regulating and inflammation‐modulatory artificial enzyme, Metazyme, was developed, which exhibited dual lactate oxidase (LOX)‐like and catalase (CAT)‐like activities to oxidize lactate and decompose hydrogen peroxide (H_2_O_2_), enabling efficient oxygen recycling. Embedding Metazyme into a rod‐shaped microgel matrix yielded MetaRgel, a localized and sustained catalytic platform for wound microenvironment modulation. The MetaRgel reduced lactate and reactive oxygen species (ROS) levels, relieved hypoxia, and downregulated glycolysis‐related enzymes such as pyruvate kinase (PKM2) and pyruvate dehydrogenase kinase isoform 1 (PDK1) in vitro and thereby suppressed the pro‐inflammatory cytokine interleukin‐6 (IL‐6) and the fibrotic mediator transforming growth factor‐β1 (TGF‐β1). In a rat full‐thickness wound model in vivo, the MetaRgel significantly accelerated healing, enhanced granulation and collagen organization, and notably reduced glycolytic enzyme activity as well as the fibrotic marker α‐smooth muscle actin (α‐SMA) and hypoxia‐inducible factor 1α (HIF‐1α). The targeting of lactate‐centered metabolic dysregulation with cascade artificial enzymes offers a promising approach to interrupt inflammation‐metabolism‐fibrosis crosstalk and promote scarless skin regeneration.

## Graphical abstract

A bifunctional lactate oxidase‐like and catalase‐like artificial enzyme (Metazyme) is integrated into a rod‐shaped microgel (MetaRgel) to enable cascade lactate oxidation and oxygen regeneration. By reprogramming the wound metabolic microenvironment, MetaRgel alleviates excessive lactate accumulation, oxidative stress, hypoxia, and inflammation, thereby suppressing fibrotic signaling, disrupting the metabolism‐inflammation‐fibrosis crosstalk, and ultimately promoting accelerated, scarless skin regeneration.

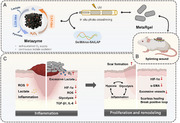

## 1 Introduction

Aberrant wound healing following skin injury often results in persistent inflammation and fibrotic scar formation, underpinned by metabolic reprogramming and dysregulated immune activation in the local microenvironment [1, 2]. Tissues typically experience hypoxia post injury, which triggers glycolytic activation (Warburg effect) and leads to excessive lactate accumulation [3, 4]. Clinical studies have reported that the lactate concentration in wound exudates can reach a median level of 21.03 mM (5.58–80.40 mM) [5], which is significantly higher than that in normal tissue (around 1.5–3 mM) [6]. Beyond its role as a glycolytic byproduct, the lactate functions as a complex signaling molecule that actively modulates wound healing. Transient lactate elevation in the early phase of tissue repair has been reported to support angiogenesis, macrophage recruitment, and re‐epithelialization by serving as both an energy substrate and a pro‐angiogenic signal [7]. In contrast, sustained and excessive lactate accumulation under prolonged hypoxia and unresolved inflammation drives pathological signaling cascades, including HIF‐1α stabilization, thereby establishing a metabolic‐inflammatory feedback loop [8, 9]. Lactate also promotes reactive oxygen species (ROS) production, induces fibroblast metabolic reprogramming toward a glycolysis‐dependent phenotype, and enhances collagen and extracellular matrix (ECM) synthesis, accelerating scar formation [10]. Moreover, recent studies have identified lactate as a donor for histone lysine lactylation, which directly activates the transcription of profibrotic genes such as collagen type I alpha 1 chain (COL1A1), further amplifying ECM deposition [11]. Therefore, dysregulated and persistent lactate accumulation is not only a hallmark of metabolic dysregulation and inflammation amplification, but also a key mediator of fibroblast reprogramming and ECM overproduction. Targeting lactate metabolism and its downstream pathways holds promise as a therapeutic strategy to modulate glycolysis and reverse fibrotic scarring. Current antifibrotic interventions targeting lactate mainly focus on glycolysis inhibitors (e.g., dichloroacetate, 2‐deoxyglucose, and oxamate) and monocarboxylate transporter (MCT) blockers that reduce lactate production or blocking its transport [12]. However, these systemic agents suffer from poor tissue specificity, potential metabolic toxicity, and limited efficacy in hypoxic microenvironments, underscoring the need for localized and sustainable lactate‐modulating approaches [12, 13].

To regulate metabolism and the inflammatory microenvironment, the construction of a cascade catalytic system based on lactate oxidase (LOX) and catalase (CAT) has emerged as a promising metabolic intervention strategy [14, 15, 16]. Natural LOX catalyzes the oxidation of lactate to pyruvate, producing hydrogen peroxide (H_2_O_2_) [17]. However, its activity relies on oxygen and is easily reduced under acidic and hypoxic conditions, limiting its effectiveness in hypoxia or fibrotic microenvironments [18]. In contrast to lactate, pyruvate has been shown to primarily drive fibroblasts toward an oxidative phosphorylation (OXPHOS)‐dominated metabolic state, without inducing a concomitant increase in glycolytic demand [9]. The hypoxia or fibrotic environments also feature high levels of ROS, especially H_2_O_2_, which aggravates oxidative stress [19]. CAT‐like enzymes can help break down H_2_O_2_ into water and oxygen, reducing ROS damage while providing oxygen to support LOX function. Since lactate removal depends on oxygen, combining LOX‐like and CAT‐like activities in a single system is essential. Such bifunctional artificial enzymes enable continuous lactate oxidation, ROS elimination, and local oxygen generation, which form a self‐sustaining catalytic loop that addresses high lactate, hypoxia, and oxidative stress. To overcome these limitations, artificial enzymes have been developed as enzyme‐mimetic catalysts with tunable structure, robust stability, and multifunctionality. Therefore, artificial enzymes with integrated LOX‐like and CAT‐like activities offer dual modulation of lactate metabolism and oxidative stress, forming a cascade catalytic system with therapeutic potential for fibrotic conditions in lactate‐enriched, hypoxic environments. On the other hand, effective localization and retention of artificial enzymes within the wound microenvironment is essential to achieve sustained therapeutic efficacy in wound settings. In this design, the microgel scaffold functions as a structural and functional matrix that retains Metazyme at the wound site, enabling continuous catalytic regulation of the local microenvironment. Our previous work demonstrated that rod‐shaped microgels, compared to bulk hydrogels, possess an interconnected porous structure that facilitates gas exchange and exudate absorption, making them especially suitable for dynamic wound environments [20].

**FIGURE 1:**
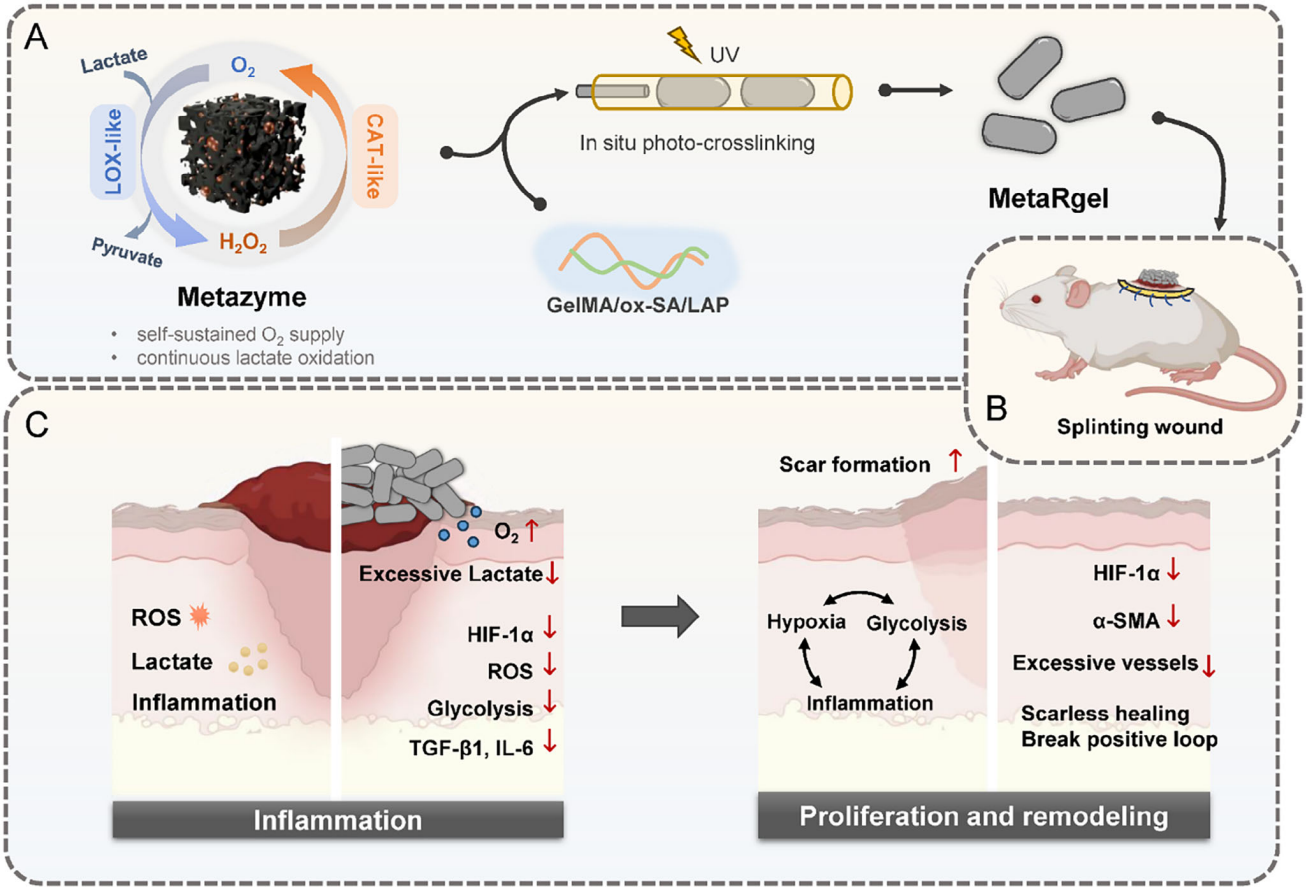
Schematic illustration of Metazyme‐loaded microgel (MetaRgel) for metabolic reprogramming and scarless wound healing. (A) The Metazyme exhibits bifunctional LOX‐like and CAT‐like mimetic activities, enabling cascade lactate oxidation and oxygen regeneration in situ. The Metazyme is homogeneously mixed with the methacrylated gelatin (GelMA)/oxidized sodium alginate (ox‐SA)/LAP prepolymer solution and directly encapsulated during the photopolymerization in situ within a microfluidic system to form rod‐shaped microgels (MetaRgel). (B) MetaRgel was topically applied to full‐thickness wound in a splinting wound model. (C) By reprogramming the local metabolic microenvironment, the MetaRgel promotes scarless wound healing by disrupting the pathological cycle of metabolic dysregulation, inflammation, and hypoxia through localized metabolic reprogramming. Created in BioRender (2026). https://BioRender.com/8dfiqrv.

In this study, the key finding is to develop a cascade Co/Mn‐based artificial enzyme with LOX‐like and CAT‐like activities, which is embedded within a rod‐shaped microgel scaffold to achieve localized and sustained catalytic regulation of the wound microenvironment. This platform enables coordinated regulation of lactate and ROS levels, thereby modulating glycolytic activity, cytokine expression, and fibroblast activation, ultimately promoting wound healing while suppressing fibrotic scarring (Figure 1). By simultaneously addressing the metabolic reprogramming and oxidative stress, our materials‐based approach would modulate the metabolism‐immunity‐fibrosis axis and break this positive feedback loop, offering a promising strategy for scarless wound healing as demonstrated by animal experiments.

## 2 Results and Discussion

### 2.1 Structural Characterization of MnPBA and Metazyme

**FIGURE 2:**
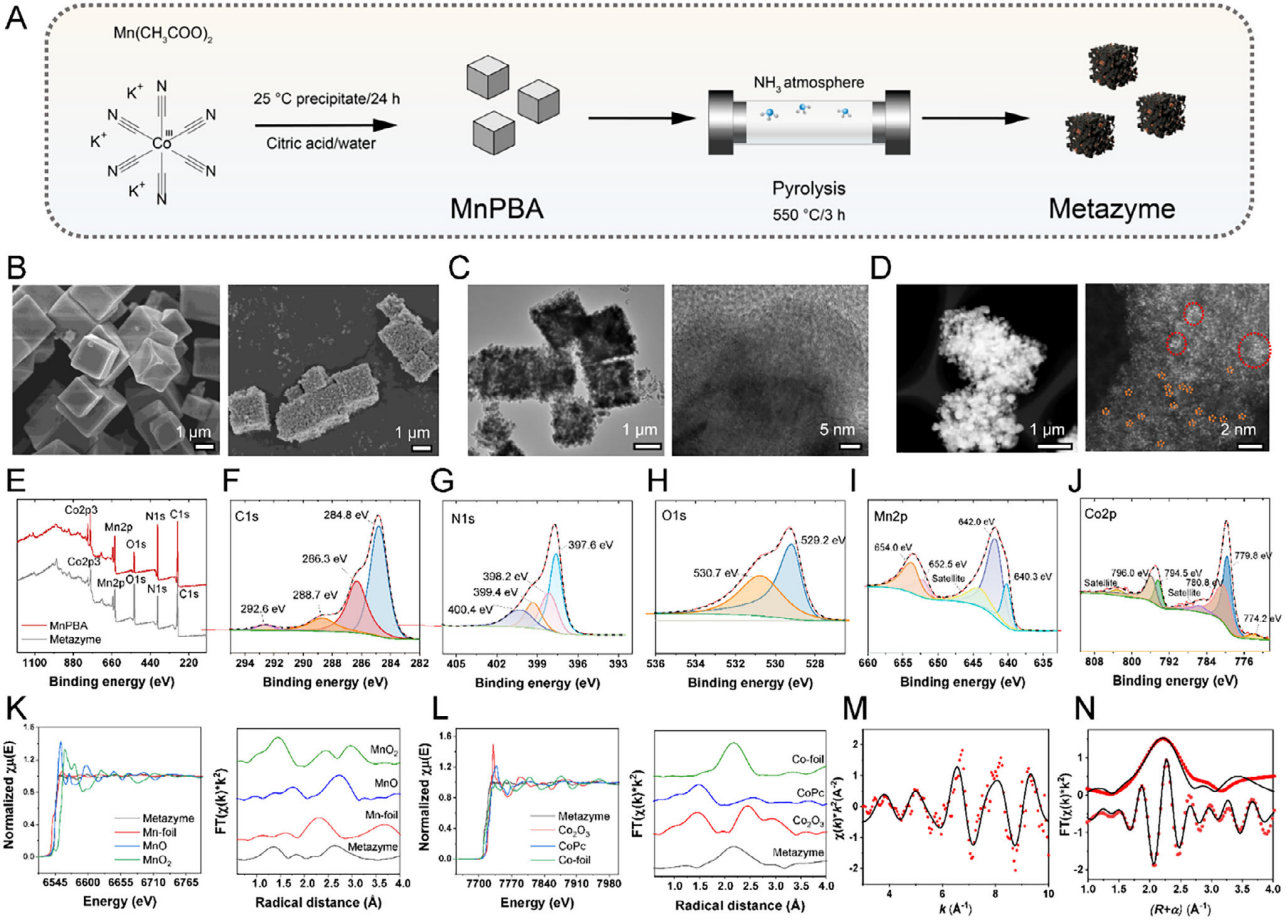
Preparation and characterization of Metazyme. (A) Schematic illustration of Metazyme synthesis via co‐precipitation of MnPBA, followed by pyrolysis under NH_3_ atmosphere. (B) SEM images showing the morphology of MnPBA (cubic crystals, left) and Metazyme (porous particles, right). (C) TEM images of Metazyme displaying its rough and porous structure. (D) AC‐STEM and high‐resolution images revealing atomically dispersed metal sites. Bright spots (orange) denote single metal atoms, while red circles indicate small clusters. (E) XPS survey spectra of MnPBA and Metazyme. High‐resolution XPS spectra of Metazyme: (F) C 1s (C = O, C–N, C–C, and C–metal), (G) N 1s (N–O, N–H, N–metal), (H) O 1s (O–metal and O–H), (I) Mn 2p (Mn^2+^/Mn^3+^ with satellites indicative of Mn–O), and (J) Co 2p (Co^2+^/Co^3+^ in Co–N), confirming heteroatom incorporation and metal oxidation states. (K) Mn K‐edge XANES (left) and EXAFS spectra (right) compared with standard references. (L) Co K‐edge XANES (left) and EXAFS spectra (right) compared with standard references. (M) Co K‐edge EXAFS (points) and the curve‐fit (line) for Metazyme, shown in k^2^ weight k space. (N) Co K‐edge EXAFS (points) and the curve‐fit (line) for Metazyme, shown R space (FT magnitude and imaginary component). The data are k^2^‐weighted and not phase‐corrected.

Prussian blue analogues (PBAs) have gained considerable attention in tissue regeneration due to their ease of synthesis, tunable structure, and intrinsic catalytic activity [21]. Thermal treatment under ammonia (NH_3_) atmosphere can reorganize atomic arrangements, thereby conferring new catalytic properties. Previous studies have demonstrated that pyrolysis of Co‐MOFs in ammonia yields Co_x_N structures, where nitrogen centers mimic flavin mononucleotide (FMN) or histidine catalytic sites, and facilitate C–H bond cleavage in lactate [22, 23, 24]. In this study, manganese‐based PBA (MnPBA) was pyrolyzed under NH_3_ at 550°C for 3 h, resulting in a novel artificial enzyme with LOX and CAT‐like activities, herein referred to as Metazyme (Figure 2A).

Scanning electron microscopy (SEM) revealed a morphological transition from well‐defined cubic particles (average size ∼1.5 ± 0.3 µm) in MnPBA to roughened surfaces with visible indentations and channel‐like structures after pyrolysis (Figure 2B). Transmission electron microscopy (TEM) further showed that the NH_3_ etching induced a hollow interior, along with mesopores and micropores on the surface. High‐magnification TEM images displayed parallel lattice fringes, which are indicative of partial graphitization (Figure 2C). This conclusion was further supported by Raman spectroscopy, where characteristic D and G bands appeared at 1340 and 1590 cm^−1^, respectively (Figure S1). Aberration‐corrected scanning transmission electron microscopy (AC‐STEM) revealed heterogeneous metal distribution in the Metazyme, with metal clusters of varying sizes. At higher magnification, discrete bright spots (orange circles) resembling atomically dispersed species were observed, suggesting the presence of single‐atom active sites, while larger domains (red circles) indicate small clusters (Figure 2D). The coexistence of atomic sites and small clusters may collectively enhance catalytic performance by balancing activity and stability to optimize reaction pathways, lower energy barriers, and thereby accelerate reaction kinetics [25, 26].

X‐Ray diffraction (XRD) was used to further confirm the structural evolution from the MnPBA precursor to the final Metazyme (Figure S2). The MnPBA exhibited sharp diffraction peaks consistent with a typical Prussian blue analog structure, indicating high crystallinity. After pyrolysis, the Metazyme showed significantly weakened and broadened peaks, suggesting a transition to a partially amorphous or nanocrystalline structure. To identify the crystalline phases, the XRD pattern of Metazyme was compared with standard references, including MnO (PDF#07‐0230), MnO_2_ (PDF#43‐1455), Co_4_N (PDF#41‐0943), and graphite (PDF#41‐1487) (Figure S3). Characteristic peaks corresponding to Metazyme were observed to analyze its chemical states and structural features. Distinct peaks matching MnO and MnO_2_ were also detected, likely due to surface oxidation. Minor peaks corresponding to Co_4_N were observed, confirming the presence of this structure. Broad signals from graphitic carbon indicate partial carbonization of the organic framework during pyrolysis.

X‐Ray photoelectron spectroscopy (XPS) was used to probe the elemental compositions and chemical states of MnPBA and Metazyme. The survey spectra confirmed the presence of Co, Mn, O, and N in both samples (Figure 2E). In the high‐resolution C 1s spectrum of Metazyme (Figure 2F), peaks at 284.8, 286.3, 288.7, and 292.6 eV correspond to C–Metal, C–C, C–N, and C = O bonds, respectively, indicating a complex carbon environment [22]. The N 1s spectrum (Figure 2G) exhibits a peak at 397.6 eV assigned to N–Co in the Co_4_N phase, along with peaks at 398.2 and 399.4 eV attributed to pyrrolic N (N–H) terminal groups formed during ammonia thermal treatment [22]. The peak at 400.4 eV corresponds to N–O species, likely resulting from surface oxidation of Co_4_N upon air exposure [27]. In the O 1s spectrum (Figure 2H), peaks at 529.2 and 530.7 eV indicate O–metal bonds and adsorbed hydroxyl or oxygen species, respectively, confirming surface oxidation [28]. The Mn 2p spectrum (Figure 2I) displays two main peaks at 642 eV (Mn 2p^3/2^) and 654 eV (Mn 2p^1/2^), which are characteristic of Mn^2+^ in MnO and consistent with previous reports [29, 30]. The observed spin‐orbit splitting of 12 eV further supports this assignment [31]. The Co 2p spectrum (Figure 2J) shows peaks at 780.8 and 796.0 eV, corresponding to Co–N bonds in the Co_4_N/C structure, [22] indicating successful nitrogen incorporation into the cobalt lattice.

To investigate the local coordination environment of metal centers, X‐ray absorption near‐edge structure (XANES) and extended X‐ray absorption fine structure (EXAFS) analysis were performed. The Mn K‐edge XANES spectrum of Metazyme closely matches that of MnO, suggesting that Mn remains mostly in the +2 oxidation state (Figure 2K). EXAFS data revealed three dominant peaks below 4 Å at ∼1.3, ∼1.8, and ∼2.6 Å, aligning well with those observed in MnO and MnO_2_, indicating preserved Mn–O coordination environments. At the Co K‐edge (Figure 2L), the Metazyme exhibited a dominant peak at ∼2.2 Å in the EXAFS spectrum, indicating Co–Co bonding as the primary coordination structure. To quantitatively assess the local structure, EXAFS fitting was performed using Artemis software (Figure 2M,N). A three‐path model containing Co–N and Co–Co scattering paths was employed to fit the R‐space data. The fit reproduced the key features of the |*χ*(*R*)| spectrum, with only minor deviations at higher R values. The energy shift (Δ*E*_0_) converged to 5.501 ± 0.004 eV, and coordination numbers remained within physically reasonable limits. The R‐factor of 0.12 is within an acceptable range, though slightly higher than ideal fits, which may reflect structural disorder and the presence of unmodelled multiple scattering contributions. Detailed fitting parameters are summarized in Table 1. Notably, the fitted coordination numbers for Co–Co paths (N2 ≈ 3.3 and N3 ≈ 2.9) were markedly smaller than the theoretical values expected for bulk Co_4_N (12 and 6, respectively), while the Co–N coordination was fixed to 2. These results imply that, in addition to the crystalline Co_4_N framework, a fraction of Co atoms exists in a highly dispersed state lacking extended Co–Co interactions. This supports the presence of isolated Co–N_x_ moieties, possibly embedded within a disordered carbon/nitrogen matrix or forming defective surface terminations. This structural interpretation is consistent with the AC‐STEM images, where discrete bright spots and bright clusters were observed. These atomic‐scale features, which appear as individual high‐Z atoms on a relatively light‐element background, can be attributed to single Co atoms stabilized by nitrogen coordination.

**TABLE 1:** Curvefit parameters^a^ for Co K‐edge EXAFS for Metazyme.

Path	Reff^b^/Å	N	R/Å	$\sigma^{2}$/Å^2^
Co−N	1.86	2.0 (set)	2.12 ± 0.06	0.01 ± 0.004
Co−Co1	2.63	3.27 ± 0.48	2.48 ± 0.01	0.003 (set)
Co−Co2	3.73	2.92 ± 1.57	3.88 ± 0.04	0.003 (set)

^a^ R‐factor: 0.12. S_0_^2^ was fixed as 0.78. Δ*E*_0_ was refined as a fit parameter, returning a value of 5.501 ± 0.004 eV. Data range: 3.0 ≤ k ≤ 10.0 (Å^−1^), 1.0 ≤ R ≤ 4.0 (Å). The number of variable parameters was 7, out of a total of 14.0 independent points.

^b^ The distances for Co−N and Co−Co paths are based on the crystal structures of Co_4_N. Debye‐Waller factors $\left(\sigma^{2}\right)$ were fixed based on reasonable values from a similar system (Co−Co path) [32].

Collectively, these results demonstrate that the pyrolysis process transformed MnPBA into a hollow, porous, partially graphitized structure with dispersed Co and Mn active sites. The incorporation of nitrogen and the formation of Co_4_N and MnO/MnO_2_ phases, along with single‐atom dispersion, provide a strong structural basis for the dual enzymatic activity of Metazyme, supporting its potential applications in metabolic regulation and tissue regeneration.

### 2.2 Catalytic Abilities of Metazyme

To identify the most effective artificial enzyme for lactate oxidation, its metal center was optimized. By varying the precursor metal sources, a series of artificial enzymes with different central metal ions were synthesized and pyrolyzed, designated as ZnN, MnN (Metazyme), CuN, and CoN. These artificial enzymes were incubated with lactate solution to measure the lactate consumption (Figure S4). Among them, the Mn‐centered Metazyme exhibited the highest lactate consumption, indicating its superior catalytic activity toward lactate. To further confirm the LOX‐like activity of Metazyme, the generation of pyruvate, the oxidative product of lactate, was quantitatively assessed (Figure S5).

**FIGURE 3:**
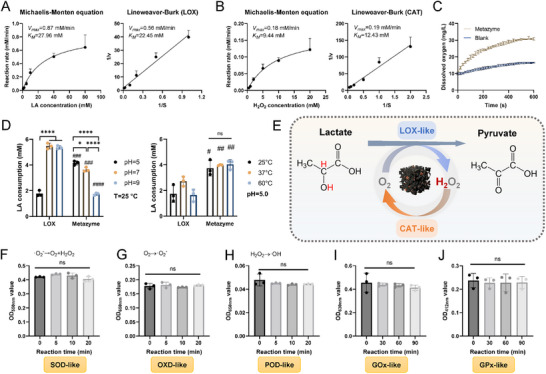
Enzymatic behavior and substrate specificity of Metazyme. (A,B) Michaelis‐Menten and Lineweaver‐Burk plots showing the LOX‐like (A) and CAT‐like (B) catalytic activities of Metazyme, with calculated *V_max_* and *K_m_* values based on lactate and H_2_O_2_ as substrates, respectively. (C) Time‐dependent dissolved oxygen generation confirming the oxygen‐recycling ability of Metazyme compared to blank control. (D) Comparison of lactate (LA) consumption by natural LOX and Metazyme under different pH and temperature conditions, indicating enhanced stability and activity of Metazyme across physiological environments. (E) Scheme of the cascade reaction catalyzed by Metazyme: lactate oxidation (LOX‐like) and H_2_O_2_ decomposition (CAT‐like), enabling continuous oxygen recycling. (F‐J) Evaluation of substrate selectivity and side enzyme‐like activities. The Metazyme showed negligible activity in SOD‐like (F), OXD‐like (G), POD‐like (H), GOx‐like (I), and GPx‐like (J) assays, indicating good substrate specificity toward lactate and H_2_O_2_. Data are presented as mean ± SD, n = 3. ns = not significant, *p < 0.05, and ****p < 0.0001 (one‐way ANOVA between selected groups). #*p* < 0.05, ##*p* < 0.01, ###*p* < 0.001, and ####*p* < 0.0001 (*t*‐test between natural enzymes and Metazyme under the same condition).

The kinetic behavior of Metazyme under room temperature in lactate oxidation was further investigated, revealing a strong correlation between the lactate consumption and lactate concentration, and the reaction followed typical Michaelis‐Menten kinetics, with good linearity in Lineweaver‐Burk plots (Figure 3A). The maximum initial reaction rates (*V_max_*) were calculated as 0.87 mM min^−1^ (Michaelis‐Menten) and 0.56 mM min^−1^ (Lineweaver‐Burk), while the corresponding Michaelis constants (*K_M_*) were 27.96 mM (Michaelis‐Menten) and 22.45 mM (Lineweaver‐Burk), respectively, indicating high substrate affinity and catalytic efficiency. The apparent turnover number (*k*_*cat*,*app*_) of the Metazyme was calculated by normalizing the maximum reaction rate (*V_max_*) to the total molar concentration of metal species [33], assuming that all metal species contribute as potential catalytic centers. Based on the ICP‐MS‐derived Mn and Co contents (3.8 wt% and 2.6 wt%, respectively) and the Metazyme dosage (100 µg mL^−1^) used in the kinetic assays, the *k*_*cat*,*app*_ value of LOX‐like activity was determined to be 0.128 s^−1^ (Michaelis‐Menten) and 0.082 s^−1^ (Lineweaver‐Burk). Accordingly, the catalytic efficiency (*k*_*cat*,*app*_/*K_M_*) of the Metazyme was calculated to be 4.58 M^−1^ s^−1^(Michaelis‐Menten) and 3.67 M^−1^ s^−1^ (Lineweaver‐Burk). To assess the dosage dependence, the lactate oxidation kinetics was additionally measured at 50 µg mL^−1^ Metazyme (Figure S6), yielding *K_M_* = 27.08 mM and *k*_*cat*,*app*_ = 0.068 s^−1^ (Lineweaver‐Burk), which are of the same order of magnitude as those obtained at 100 µg mL^−1^.

In addition to LOX‐like activity, the Metazyme exhibited catalase (CAT)‐like behavior. The kinetic parameters for H_2_O_2_ decomposition were determined (Figure 3B), with *V_max_* values of 0.18 mM min^−1^ (Michaelis‐Menten) and 0.19 mM min^−1^ (Lineweaver‐Burk), and *K_M_* values of 9.44 mM (Michaelis‐Menten) and 12.43 mM (Lineweaver‐Burk), respectively, suggesting efficient catalytic activity toward H_2_O_2_. Based on the total molar concentration of metal species, the *k*_*cat*,*app*_ for H_2_O_2_ decomposition was calculated to be 0.027 s^−1^ (Michaelis‐Menten) and 0.028 s^−1^ (Lineweaver‐Burk). The corresponding catalytic efficiency (*k*_*cat*,*app*_/*K_M_*) was 2.81 M^−1^ s^−1^ (Michaelis‐Menten) and 2.24 M^−1^ s^−1^ (Lineweaver‐Burk), respectively. At a reduced Metazyme dosage of 50 µg mL^−1^, the CAT‐like kinetics gave *K_M_* = 14.27 mM and *k*_*cat*,*app*_ = 0.038 s^−1^ (Lineweaver‐Burk), further supporting the stability of the intrinsic metal‐normalized catalytic activity (Figure S7).

To validate its oxygen‐generating capacity, a dissolved oxygen probe was used to monitor O_2_ release during H_2_O_2_ decomposition (Figure 3C). Rapid oxygen evolution was observed within a short period of time, confirming Metazyme's ability to efficiently decompose H_2_O_2_ and continuously produce oxygen. This self‐sustained oxygen supply supports the lactate oxidation process and establishes a dual‐enzyme catalytic cycle.

To benchmark its performance, the Metazyme with commercial natural LOX under different reaction conditions was compared. At 25°C, the lactate consumption was assessed at pH 5, 7, and 9 (Figure 3D). The Metazyme exhibited optimal catalytic activity in acidic conditions, with activity decreasing as pH increased. This trend may be attributed to the enhanced protonation and favorable charge distribution at acidic pH, which promotes substrate binding and electron transfer. Notably, at pH 5, the Metazyme showed even higher catalytic efficiency than natural LOX, highlighting its advantage as a lactate oxidation catalyst under mildly acidic conditions. Furthermore, the lactate consumption was assessed at pH 5 across 25°C, 37°C, and 60°C (Figure 3D). The natural LOX showed strong temperature sensitivity, with peak activity at 37°C and substantial loss at 60°C, revealing its thermal instability. In contrast, the Metazyme maintained stable activity across all temperatures, showing no clear temperature dependence. More importantly, it consistently outperformed natural LOX under all tested conditions, demonstrating excellent thermal stability and environmental adaptability suitable under mildly acidic conditions. A previously proposed Ping‐Pong mechanism for Co_4_N/C nanozymes is likely applicable to our Metazyme [22]. In this pathway, the α‐C−H and α‐C−OH protons from lactate are transferred to Co_4_N, forming pyruvate and a Co_4_NH_2_ intermediate, which is then re‐oxidized by O_2_ to generate H_2_O_2_ and regenerate the Co_4_N active site [22].

The CAT‐like activity of the Metazyme was also evaluated by comparing its H_2_O_2_ decomposition performance with that of commercial natural CAT under different pH and temperature conditions (Figure S8). At 25°C, H_2_O_2_ consumption was assessed at pH 5, 7, and 9. Both the natural CAT and Metazyme exhibited pronounced pH dependence, with optimal activity at mildly basic (pH 9) and a marked decline under mildly acidic (pH 5) for Metazyme. The temperature dependence of CAT‐like activity was further examined at pH 9 over a range of 25, 37, and 60°C. The natural CAT displayed strong thermal sensitivity, with peak activity at 37°C and a substantial loss of activity at 60°C, indicative of enzyme denaturation. By contrast, the Metazyme preserved nearly constant H_2_O_2_ decomposition efficiency across all tested temperatures, exhibiting no statistically significant temperature dependence. Therefore, the Metazyme integrates LOX‐like and CAT‐like activities into a single platform to enable a pH‐adaptive cascade for coordinated regulation of lactate and H_2_O_2_. Under mildly acidic conditions, the Metazyme exhibits dominant LOX‐like activity, leading to rapid lactate oxidation. Importantly, the conversion of lactate into pyruvate is accompanied by a net reduction in acid burden, which contributes to the alleviation of lactate‐associated acidosis and a gradual elevation of local pH [6]. As the microenvironment shifts toward neutral pH, the CAT‐like function of the Metazyme becomes increasingly strengthened. Therefore, the CAT‐like activity of the Metazyme enables efficient decomposition of the generated H_2_O_2_ into O_2_, establishing a self‐sustained O_2_ cycle that continuously fuels the lactate oxidation reaction (Figure 3E). Through this naturally closed, time‐resolved cascade, the Metazyme dynamically matches the evolving wound microenvironment, achieving coordinated control over lactate metabolism, redox homeostasis, and oxygen availability.

To evaluate the catalytic stability of Metazyme, cyclic catalysis tests were conducted for both the LOX‐like and CAT‐like activities using the same batch of materials. The catalytic performance remained consistent across ten consecutive cycles in both reactions (Figure S9). The LOX‐like activity was well maintained over ten consecutive cycles, while a slight decrease in the CAT‐like activity was observed, which is likely attributable to partial surface passivation or active‐site blockage caused by accumulated reaction intermediates and repeated exposure to H_2_O_2_ under oxidative conditions [34]. To evaluate the storage stability of Metazyme under physiologically relevant aqueous conditions, the catalytic activities of Metazyme after incubation in PBS for 7 d were examined and compared with those of freshly prepared samples (0 d). As shown in Figure S10, no significant decrease in either LOX‐like or CAT‐like catalytic activity was observed after PBS incubation. To further evaluate the catalytic stability of Metazyme in physiologically relevant environments, its LOX‐like and CAT‐like activities were examined in PBS, simulated body fluid (SBF), and SBF supplemented with 1 mg mL^−1^ bovine serum albumin (SBF + BSA). As shown in Figure S11, the Metazyme exhibited comparable catalytic activities in PBS and SBF, indicating that variations in ionic composition and inorganic salt content do not noticeably affect its catalytic performance. In contrast, a moderate decrease in catalytic activity was observed in the presence of proteins. This reduction is likely attributable to protein adsorption onto the surface of Metazyme, which may partially block or shield the exposed catalytic metal sites, thereby limiting substrate accessibility.

To further assess the substrate specificity of Metazyme, the activities in other enzyme‐mimetic reactions were evaluated. The results revealed negligible activity in superoxide dismutase (SOD)‐like (Figure 3F), oxidase (OXD)‐like (Figure 3G), peroxidase (POD)‐like (Figure 3H), glucose oxidase (GOx)‐like (Figure 3I), and glutathione peroxidase (GPx)‐like (Figure 3J) reactions, indicating that the Metazyme exhibits high substrate selectivity and catalytic specificity toward lactate and H_2_O_2_.

In summary, the Metazyme demonstrates not only excellent catalytic efficiency for lactate oxidation but also robust CAT‐like activity, enabling continuous oxygen generation to support a self‐sustained oxidation cycle. Its superior performance over natural LOX in acidic and high‐temperature environments, combined with high substrate specificity and dual‐function catalytic behavior, highlights its potential as a versatile and robust artificial enzyme for metabolic regulation in complex physiological conditions. These advantageous properties are likely attributed to its partially graphitized carbon framework, the presence of synergistic Co−N and Mn−O active sites, and its structurally stable nanoarchitecture.

### 2.3 Preparation of Rod‐Shaped Microgels for Metazyme Encapsulation

**FIGURE 4:**
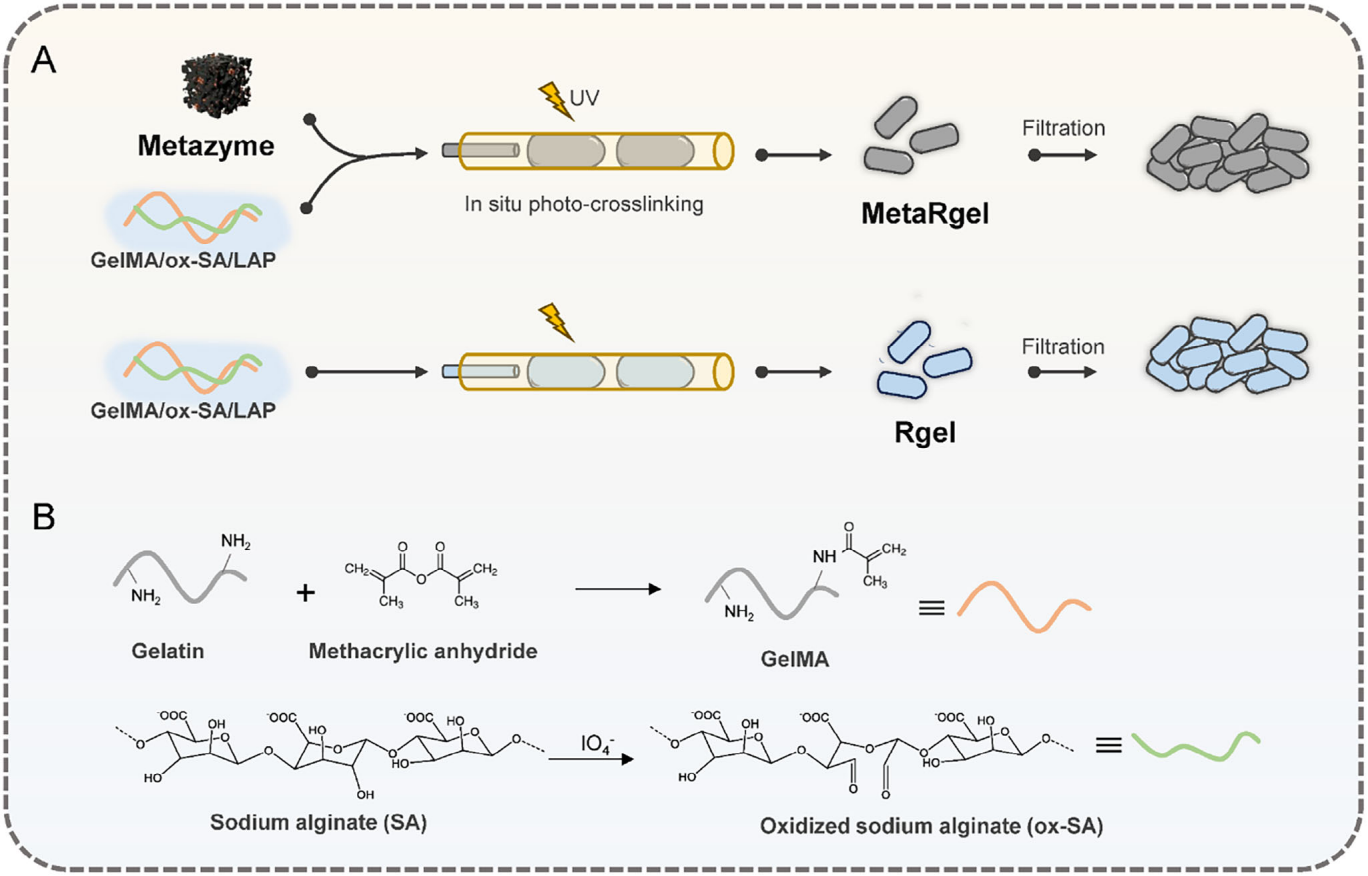
Schematic illustration of the preparation of MetaRgel and Rgel. (A) Fabrication of rod‐shaped microgels. The Metazymes were mixed with the GelMA/ox‐SA/LAP solution and subjected to photo‐crosslinking in situ within a microfluidic system, followed by filtration to obtain the Metazyme‐loaded microgels (MetaRgel). The control microgels (Rgel) were prepared using the same procedure without Metazymes. (B) Gelatin was modified with methacrylic anhydride (MA) to obtain methacrylated gelatin (GelMA), while sodium alginate (SA) was oxidized by sodium periodate to yield oxidized sodium alginate (ox‐SA).

To enable biological applications, the Metazyme was embedded into a biocompatible microgel carrier composed of methacrylated gelatin (GelMA) and oxidized sodium alginate (ox‐SA), crosslinked via lithium phenyl‐2,4,6‐trimethylbenzoylphosphinate (LAP)‐initiated photopolymerization (Figure 4). The Metazymes were first dispersed into the hydrogel prepolymer solution, which was subsequently injected into the microfluidic device and crosslinked in situ under UV irradiation, resulting in Metazyme‐loaded microgels. The unloaded rod‐shaped microgels were also prepared for comparison, which were designated as Rgel, while the Metazyme‐loaded were referred as MetaRgel. GelMA and ox‐SA were selected due to their excellent biocompatibility and abundant functional groups, which not only support cell‐material interactions but also facilitate uniform dispersion and enhanced stability of the embedded artificial enzymes. The chemical structure of the synthesized GelMA was characterized by ^1^H nuclear magnetic resonance spectroscopy (NMR). As shown in Figure S12, the characteristic signals of the vinyl methylene protons were observed at 5.44 and 5.63 ppm, while the methyl proton peak appeared at 1.90 ppm, confirming the successful methacrylation of gelatin. The chemical structure of ox‐SA was also verified by ^1^H‐NMR (Figure S13). The microgels were fabricated using a microfluidic technique to generate a rod‐shaped structure (Figure S14). Compared with the spherical microgels, the rod‐shaped microgels have been reported to assemble into scaffolds with anisotropic and elongated pore architectures featuring larger effective pore sizes, which facilitate air permeability and mass transport within the network [20, 35]. In addition, assemblies of rod‐shaped microgels exhibit higher mechanical moduli owing to enhanced interparticle interlocking [36], thereby providing improved structural resilience in dynamic wound environments. Such properties are particularly advantageous as breathable and mechanically stable wound dressings, where sufficient oxygen diffusion and metabolite transport are critical. The stability of the MetaRgel and the retention behavior of the embedded Metazyme were assessed by incubating the MetaRgel in PBS. Flow cytometry analysis of the collected samples over a 3‐day period revealed negligible Metazyme‐associated signals, indicating minimal particle leakage from the microgel matrix (Figure S15). Owing to their high specific surface area, both the MetaRgel and Rgel exhibited a high swelling ratio in water and PBS (Figure S16). The microgels were collected and filtered for further use in vitro and in vivo. Rheological analysis revealed that the MetaRgel exhibited G′ values higher than G″ under low‐frequency conditions, indicating solid‐like behavior that maintains structural integrity (Figure S17). Under low‐strain (5%) conditions, the G′ of MetaRgel was also higher than G″; in contrast, at high strain (500%), G″ exceeded G′, suggesting enhanced flowability that facilitates extrusion (Figure S18). Comprehensive characterizations of the microgels were conducted, with detailed data provided in the Supplementary Information.

### 2.4 Dual‐Functional Activities of Metazyme‐Loaded Microgels In Vitro

First, the cytotoxicity of our materials against L929 fibroblast cells (Figure S19) was compared to the control group, indicating that the incorporation of Metazyme did not cause apparent cytotoxicity. To explore the regulatory effect of the material system on cellular homeostasis under stress conditions, L929 fibroblasts were stimulated with hypoxia, lipopolysaccharide (LPS), and lactate (LA) to simulate oxidative stress, inflammation, and metabolic dysregulation. The cellular responses across multiple dimensions were then evaluated, including ROS levels, hypoxia status, lactate metabolism, inflammatory cytokine secretion, and the expression of key glycolytic enzymes.

**FIGURE 5:**
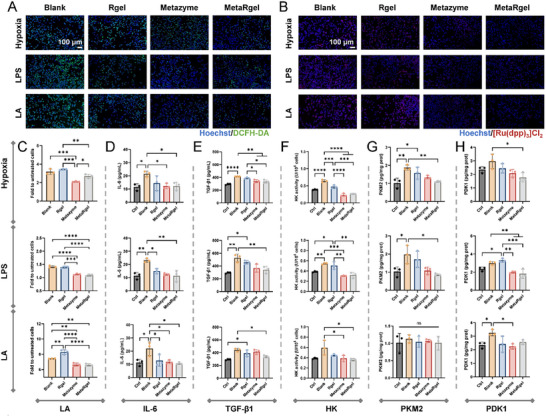
Antioxidant and metabolic regulatory effects of Metazyme and MetaRgel in L929 cells. (A) Fluorescence images of intracellular ROS levels (DCFH‐DA, green) in L929 cells under hypoxia, LPS, or lactate (LA) stimulation without (Blank) and with Rgel, Metazymes and MetaRgel, respectively. Nuclei were stained with Hoechst (blue). (B) Intracellular hypoxia levels visualized using [Ru(dpp)_3_]Cl_2_ staining (red), indicating alleviation of hypoxia in the treated groups. (C‐E) Quantification of LA (C), IL‐6 (D), and TGF‐β1 (E) levels in the cell culture supernatants under different stress conditions. (F‐H) Activities or expression levels of glycolysis‐related markers: hexokinase (HK) activity (F), PKM2 (G), and PDK1 (H), showing that the Metazyme and MetaRgel significantly downregulated the glycolytic metabolism. Data are presented as mean ± SD, n = 3. ns = not significant, *p < 0.05, **p < 0.01, ***p < 0.001, and ****p < 0.0001 (one‐way ANOVA).

2’,7’‐Dichlorodihydrofluorescein diacetate (DCFH‐DA) staining (Figure 5A) and its semi‐quantification (Figure S20A) showed intense green fluorescence in the Blank group under all three stimuli, indicating a significant increase in intracellular ROS. The Rgel group showed only mild alleviation, suggesting that the microgels alone had limited antioxidant capacity. The Metazyme group, owing to the bifunctional catalytic activity of LOX and CAT, significantly reduced ROS levels by degrading lactate and H_2_O_2_. The antioxidant effect observed in the MetaRgel group was comparable, indicating that the encapsulation of enzymes in the microgels did not impair their antioxidation function.

To further verify the effect on cellular hypoxia, the oxygen‐sensitive fluorescence probe [Ru(dpp)_3_]Cl_2_ was employed to stain L929 cells subjected to hypoxia, LPS, and lactate stimulation. In [Ru(dpp)_3_]Cl_2_ staining, the red fluorescence intensity is positively correlated with the hypoxia level: the stronger the signal, the more severe the hypoxia [37]. Under oxidative stress and mitochondrial damage, excessive ROS compromises the mitochondrial membrane potential, impairing oxygen uptake and utilization, ultimately leading to intracellular hypoxia [38]. As shown in Figure 5B and Figure S20B, the Blank group displayed strong red fluorescence under all stimuli, suggesting pronounced intracellular hypoxia. In contrast, the Metazyme group exhibited significantly reduced fluorescence, especially under lactate stimulation, indicating its capacity to alleviate hypoxia via catalytic oxygen generation. The MetaRgel group exhibited the weakest red fluorescence, demonstrating superior oxygen‐enhancing performance.

Consistent with these observations, extracellular lactate measurement (Figure 5C) showed significant lactate accumulation in the Blank and Rgel groups under all stress conditions. In contrast, both the Metazyme and MetaRgel significantly reduced the lactate levels, confirming their effective regulation of lactate metabolism.

As the end product of glycolysis, lactate is a key marker of inflammation and metabolic dysregulation [3, 9]. Its continuous accumulation exacerbates acidic microenvironments and promotes inflammatory cytokine secretion. Analysis of the inflammatory cytokines further revealed the critical role of material intervention in disrupting the metabolism‐inflammation‐fibrosis feedback loop (Figure 5D,E). Under all three stimuli, the IL‐6 and TGF‐β1 levels were significantly elevated in the Blank group, indicating activation of inflammatory and fibrotic potentials. All the Rgel, Metazyme, and MetaRgel treatments reduced the IL‐6 levels to varying extents, with MetaRgel showing the most pronounced effect. IL‐6 not only acts as a classical pro‐inflammatory cytokine but also promotes glycolytic gene expression via signal transducer and activator of transcription 3 (STAT3) activation, enhancing lactate production and forming a ROS‐inflammation‐metabolism feedback loop [39, 40, 41]. The TGF‐β1 expression was also significantly reduced after Metazyme and MetaRgel treatment, suggesting that attenuation of lactate levels and inflammation helped suppress downstream fibrotic signaling, thus contributing to the restoration of tissue homeostasis in a less fibrotic status.

To further assess the key factors in metabolic reprogramming, the expression of critical glycolytic enzymes involved in the Warburg effect was examined, including hexokinase (HK), M2‐pyruvate kinase (PKM2), and pyruvate dehydrogenase kinase 1 (PDK1). Under hypoxia, LPS, or lactate stimulation, the Blank group exhibited significantly elevated HK activity, indicating enhanced glycolytic flux and a classical Warburg phenotype (Figure 5F). The Rgel group showed limited alleviation, whereas both the Metazyme and MetaRgel markedly inhibited HK activity, especially under lactate stimulation, demonstrating potent metabolic intervention and inhibition of lactate overproduction.

Further analysis of PKM2 expression (Figure 5G) revealed significant upregulation under hypoxia and LPS stimulation, but not under lactate stimulation. As a rate‐limiting enzyme in the final step of glycolysis, PKM2 catalyzes the conversion of phosphoenolpyruvate to pyruvate, playing a central role in controlling glycolytic flux and metabolic reprogramming. Aberrant activation of PKM2 is not only a key feature of tumor‐like metabolism but is also implicated in pulmonary, hepatic, and cardiac fibrosis [8, 42, 43]. Its glycolytic‐promoting effect contributes to lactate accumulation, local acidification, fibroblast activation, and increased expression of pro‐fibrotic cytokines such as TGF‐β1, thereby forming a prototypical metabolism‐inflammation‐fibrosis feedback loop [8, 44]. Thus, effective suppression of PKM2 is not only crucial for correcting metabolic disorders but may also serve as a key target for anti‐fibrotic interventions. PKM2 expression is upregulated under hypoxic and inflammatory stress via HIF‐1α and nuclear factor kappa‐light‐chain‐enhancer of activated B cells (NF‐κB) signaling [45, 46]. However, under exogenous lactate stimulation, lactate as a downstream metabolite does not significantly affect PKM2 expression.

The PDK1 expression showed a similar trend (Figure 5H): significantly elevated in the Blank group, and markedly reduced following Metazyme or MetaRgel treatment. As a key member of the pyruvate dehydrogenase kinase family, PDK1 inhibits the pyruvate dehydrogenase (PDH) complex via phosphorylation, blocking pyruvate entry into the mitochondrial tricarboxylic acid (TCA) cycle and redirecting it toward lactate production [47]. Under pathological conditions such as hypoxia, inflammation, and high lactate levels, PDK1 is frequently overactivated and is a core driver of the metabolic shift from oxidative phosphorylation to glycolysis. Previous studies have shown that exogenous lactate can indirectly enhance PDK1 expression through activation of GPR81 (a lactate receptor), acid‐sensing channels, and PI3K/Akt/mTOR signaling pathways, further promoting glycolytic flux and lactate accumulation [48]. PDK1 is widely upregulated in metabolic disorders and fibrotic diseases. Its sustained activation exacerbates cellular metabolic burden and oxidative stress, thereby driving fibroblast activation and collagen deposition. Therefore, downregulation of PDK1 expression not only aids in restoring mitochondrial oxidative metabolism but may also help suppress subsequent fibrotic processes.

In summary, under hypoxia, LPS, or lactate stimulation, L929 cells exhibited a typical metabolism‐inflammation‐fibrosis positive feedback phenotype, characterized by ROS accumulation, lactate elevation, hypoxia, and upregulation of inflammatory and glycolytic proteins. Intervention with the Metazyme or MetaRgel disrupted this pathological loop by reducing ROS and lactate levels through catalytic lactate degradation and oxygen release, and by suppressing the expression of IL‐6, TGF‐β1, and key glycolytic enzymes. These effects jointly contribute to the restoration of cellular microenvironmental homeostasis with metabolic regulatory and anti‐fibrotic potential.

### 2.5 Enhanced Healing and Reduced Fibrotic Scarring in a Rat Full‐Thickness Wound Model

**FIGURE 6:**
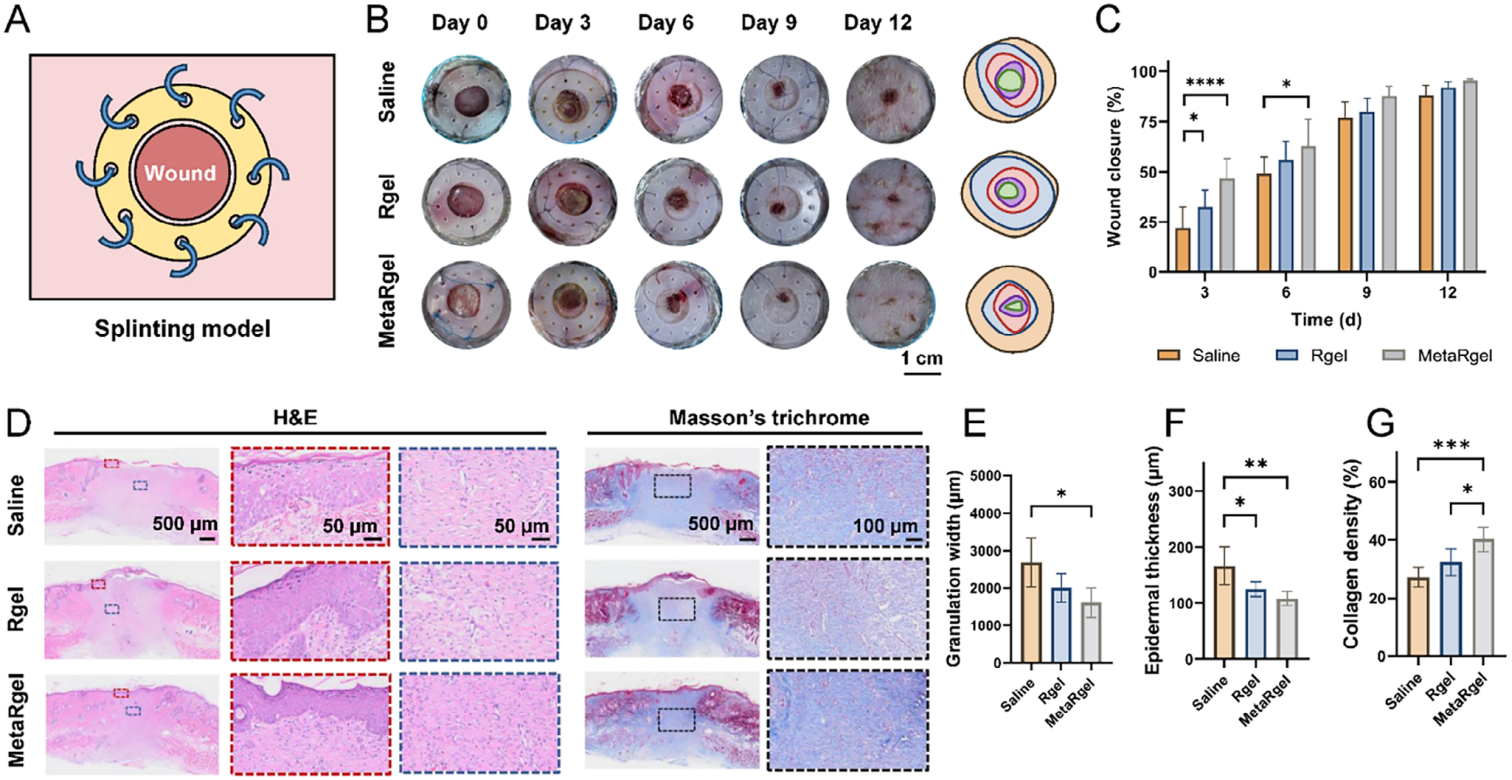
Evaluation of MetaRgel in a rat splinting full‐thickness wound model in vivo. (A) Schematic illustration of the splinting wound model used to prevent contraction and evaluate re‐epithelialization and granulation. (B) Representative wound images from saline, Rgel, and MetaRgel groups on days 0, 3, 6, 9, and 12 post‐injuries. (C) Quantitative analysis of wound closure rates over time, showing accelerated healing in the MetaRgel group. (D) H&E and Masson's trichrome staining of wound tissues at day 12, demonstrating enhanced granulation and collagen deposition in the MetaRgel group. (E–G) Semi‐quantification of histological parameters: granulation tissue width (E), epidermal thickness (F), and collagen density (G), confirming improved wound remodeling with the MetaRgel treatment. Data are presented as mean ± SD, n = 5, biologically independent samples. *p < 0.05, **p < 0.01, ***p < 0.001, ****p < 0.0001 (one‐way ANOVA).

To evaluate the wound healing efficacy in vivo, a full‐thickness excisional wound model on the dorsal skin of rats was established. A splinting ring was sutured around the wound site (Figure 6A) to minimize the natural wound contraction, thereby encouraging granulation tissue formation rather than contraction‐based closure, which more closely mimics the healing pattern in human skin and facilitates the formation of scar‐like tissue [49]. As shown in Figure 6B and quantitatively in Figure 6C, the MetaRgel treatment significantly accelerated the early wound healing. On days 3 and 6, the wound closure percentages in the MetaRgel group were markedly higher than those in both the Saline and Rgel groups (*p <* 0.05), suggesting that the MetaRgel promotes rapid granulation and epithelialization in the early inflammatory phase, owing to its antioxidative and immunomodulatory effects.

Histological analysis was performed on day 12 using hematoxylin and eosin (H&E) staining (Figure 6D). In the Saline group, epithelial regeneration remained incomplete, with loosely organized granulation tissue and an inflammatory cell‐rich environment. In contrast, both the Rgel and MetaRgel groups exhibited more mature epithelial layers and reduced inflammatory cell infiltration. Notably, the MetaRgel group showed a well‐structured epidermis with visible rete ridges and more compact underlying tissue, indicating enhanced tissue remodeling. Collagen deposition using Masson's trichrome staining was further assessed (Figure 6D). The Saline group displayed sparse and disorganized collagen fibers, whereas the MetaRgel group exhibited denser and more aligned collagen bundles, suggesting more effective matrix remodeling and scar organization. Semi‐quantitative analysis confirmed these observations (Figure 6E–G). The MetaRgel treatment significantly reduced granulation tissue width (*p <* 0.01), increased epidermal thickness (*p <* 0.05), and improved collagen density compared to the Saline and Rgel groups (*p <* 0.001). These results indicate that our MetaRgel not only promotes faster wound closure in the early stage but also enhances tissue quality in the remodeling phase by supporting collagen maturation and re‐epithelialization.

**FIGURE 7:**
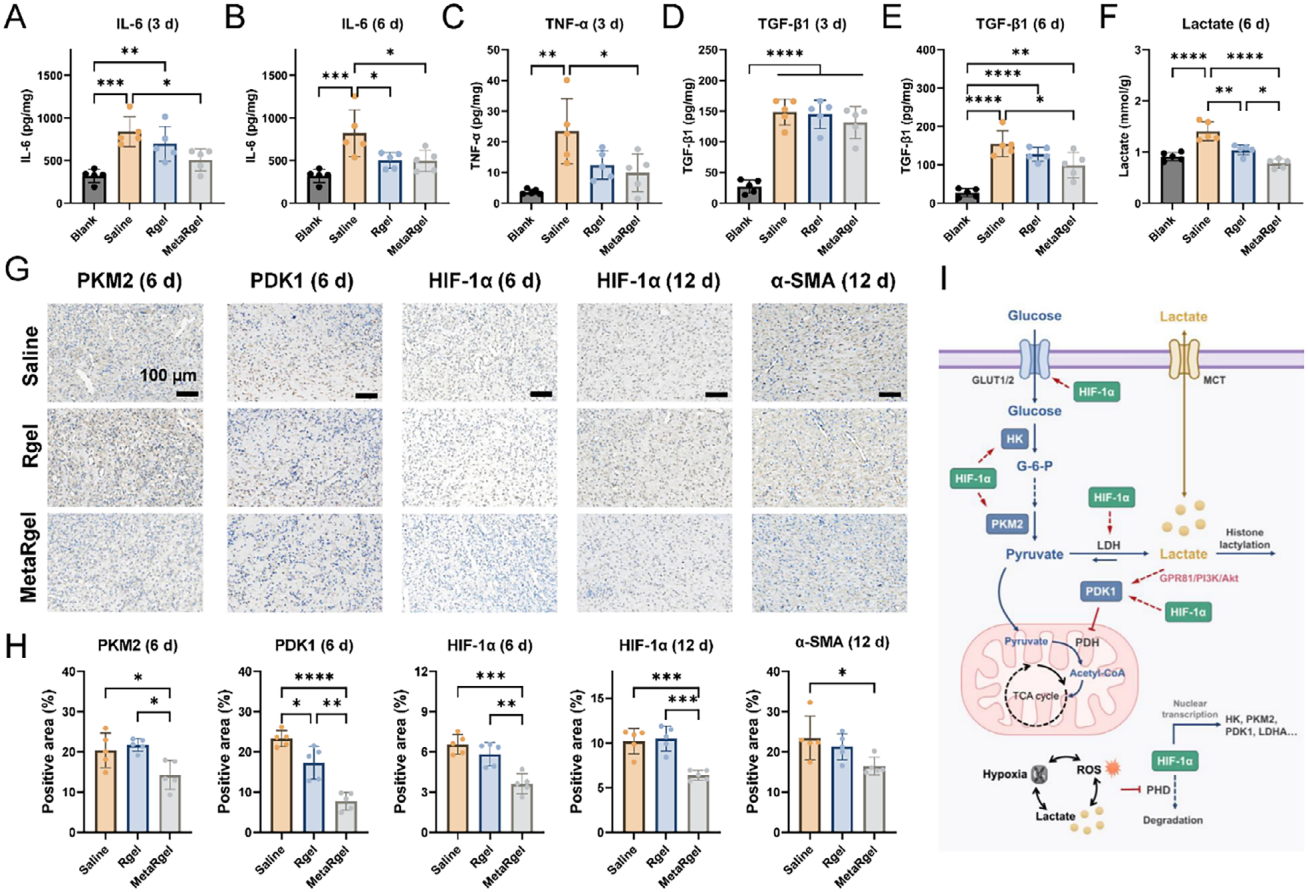
MetaRgel modulates inflammation and glycolysis‐related fibrosis pathways in vivo. (A–F) Quantification of inflammatory and fibrotic markers in wound tissues. The MetaRgel treatment significantly reduced IL‐6 (A and B), TNF‐α (C), and TGF‐β1 (D and E) levels at day 3 and day 6, as well as lactate accumulation (F), compared to the Saline and Rgel groups. (G) Representative immunohistochemical (IHC) staining images of PKM2, PDK1, HIF‐1α (day 6 and 12), and α‐SMA (day 12) in wound tissues, showing reduced glycolytic and fibrotic marker expression in the MetaRgel group. (H) Semi‐quantification of IHC‐positive areas for PKM2, PDK1, HIF‐1α, and α‐SMA, confirming significant suppression of glycolysis and hypoxia‐induced fibrosis. (I) Scheme of lactate‐mediated metabolic and signaling pathways in wound healing. The MetaRgel disrupts the lactate/HIF‐1α/glycolysis/fibrosis axis by reducing lactate and reprogramming cellular metabolism. Data are presented as mean ± SD, n = 5, biologically independent samples. *p < 0.05, **p < 0.01, ***p < 0.001, and ****p < 0.0001 (one‐way ANOVA).

To further investigate how the material system regulates inflammation, hypoxia, and metabolic stress during wound healing, the inflammatory cytokines and lactate levels in wound tissues at early time points were analyzed (Figure 7A,B). The IL‐6 levels were markedly elevated in the Saline group on days 3 and 6, indicating a prolonged inflammatory response. Both the Rgel and MetaRgel treatments reduced IL‐6 expression, with MetaRgel showing the most pronounced decrease, suggesting more effective resolution of early inflammation. Similarly, tumor necrosis factor‐α (TNF‐α), a key mediator of acute inflammation and immune cell recruitment, was significantly reduced in the MetaRgel group compared to Saline on day 3 (Figure 7C), further supporting its anti‐inflammatory potential. TGF‐β1, a cytokine involved in both inflammation resolution and fibrotic signaling, was also elevated in the Saline and Rgel groups at day 3 (Figure 7D), while was attenuated in the MetaRgel group. The difference became more pronounced at day 6 (Figure 7E), where the TGF‐β1 level in the MetaRgel group was significantly lower than that in the Saline group, indicating suppression of fibrotic activation during tissue remodeling.

The lactate content in wound tissue, as a metabolic indicator of glycolytic stress and hypoxia (Figure 7F), was markedly elevated in the Saline group on day 6. The Rgel showed moderate reduction, whereas the MetaRgel significantly decreased excessive lactate accumulation (*p <* 0.01), which in turn alleviated local metabolic stress and supported the oxygen‐generating capacities.

To further evaluate the modulation of hypoxia‐related metabolism and fibrotic signaling in vivo, immunohistochemical (IHC) staining of key proteins involved in glycolysis, hypoxia response, and myofibroblast activation was performed, followed by semi‐quantitative analysis (Figure 7G,H). On day 6, the expression of PKM2 and PDK1, two enzymes central to glycolytic reprogramming, was significantly elevated in the Saline group, but was markedly reduced in the MetaRgel group (*p <* 0.05 for PKM2; *p <* 0.001 for PDK1), indicating effective suppression of glycolytic activation in the wound microenvironment. Similarly, HIF‐1α showed strong nuclear accumulation in the Saline group at both day 6 and day 12. In contrast, the MetaRgel substantially downregulated HIF‐1α expression at both time points (*p <* 0.001), suggesting improved oxygen availability and alleviation of hypoxic stress. On day 12, the MetaRgel also significantly reduced α‐SMA‐positive area compared to the Saline group (*p <* 0.05), indicating decreased fibrotic activation during the remodeling phase.

In addition, IHC staining for CD31 was performed to evaluate neovascularization dynamics at different stages of wound healing (Figure S21). Quantitative analysis of vessel density revealed no significant differences in the numbers of CD31‐positive microvessels among the Saline, Rgel, and MetaRgel groups on day 6, indicating comparable angiogenic responses during the early proliferative phase. However, by day 12, the MetaRgel group exhibited a significantly lower vessel density compared with the Saline group (*p <* 0.05). This reduction in micro‐vessel number may be attributed to excessive lactate clearance and sustained suppression of HIF‐1α signaling, which together shift the angiogenic balance from excessive neovascular formation toward vascular pruning and maturation. During normal tissue regeneration, transiently formed microvessels are selectively regressed, preserving only functional and stable vasculature. In contrast, fibrotic and hypertrophic scar tissues are often characterized by persistent, excessive, and poorly functionalized angiogenesis [50, 51]. Therefore, the controlled reduction of CD31‐positive vessels observed in the MetaRgel group at the remodeling stage represents another aspect of attenuated fibrosis and improved tissue regeneration.

To further assess the biosafety of the MetaRgel system in vivo, systemic toxicity was evaluated by blood biochemical analysis and histological examination of major organs. Blood samples collected on day 6 were analyzed for key liver and kidney function indicators, including alanine aminotransferase (ALT), aspartate aminotransferase (AST), alkaline phosphatase (ALP), blood urea nitrogen (BUN), and creatinine (Cr). As shown in Figure S22, no significant differences were observed among the Saline, Rgel, and MetaRgel groups, indicating the absence of acute systemic toxicity. In addition, H&E staining of the harvested heart, liver, spleen, lung, and kidney revealed no apparent pathological abnormalities or inflammatory lesions in the MetaRgel‐treated groups compared with the Blank groups (Figure S23), confirming the biosafety of MetaRgel during the wound healing treatment in vivo.

In summary, our study demonstrates that the wound microenvironments characterized by hypoxia, inflammation, and metabolic reprogramming can establish a pathological positive feedback loop driven by HIF‐1α mediated transcription of glycolytic enzymes such as HK, PKM2, PDK1, and lactate dehydrogenase A (LDHA) (Figure 7I). This shift toward aerobic glycolysis leads to excess lactate accumulation, which further stabilizes HIF‐1α via inhibition of PHD and activation of related signaling. Lactate also contributes to fibrosis by inducing histone lactylation and the expression of pro‐fibrotic genes. Together, these processes sustain oxidative stress, promote myofibroblast activation, and impair regenerative healing. By catalytically degrading lactate and generating oxygen, the MetaRgel with Metazyme interrupts this pathological loop at multiple levels. These interventions reduce ROS accumulation, alleviate HIF‐1α stabilization, suppress glycolytic enzyme expression, and ultimately attenuate fibrotic signaling. The resulting modulation of the hypoxia‐glycolysis‐fibrosis axis promotes a more balanced and regenerative wound healing response, offering a promising strategy for preventing scar formation and improving tissue repair outcomes.

## 3 Conclusion

Effective regulation of wound metabolism and oxidative stress is essential for achieving scarless skin regeneration, yet remains a significant challenge in current therapeutic strategies. In this study, we developed and successfully applied a bifunctional artificial enzyme named Metazyme that is capable of simultaneously catalyzing lactate oxidation and H_2_O_2_ decomposition. By embedding Metazymes into biocompatible microgels to form the MetaRgel, we constructed a multifunctional system capable of reshaping the pathological wound microenvironment. Comprehensive experiments in vitro and in vivo demonstrated that the MetaRgel containing Metazymes effectively consumed lactate and generated oxygen, thereby alleviating intracellular ROS accumulation, inhibiting HIF‐1α stabilization, and downregulating key glycolytic enzymes such as PKM2 and PDK1. These metabolic modulations further suppressed the release of pro‐inflammatory cytokines (e.g., IL‐6 and TNF‐α), attenuated fibrotic signaling (TGF‐β1, α‐SMA), and promoted the formation of well‐organized granulation tissue and collagen matrix. In a splinting full‐thickness skin defect model on the dorsal side of rats in vivo, the MetaRgel significantly accelerated wound healing and reduced scar formation, achieving a regenerative outcome resembling scarless healing. Our findings underscore the potential of cascade artificial enzymes‐based therapy as a generalizable strategy for reprogramming the wound microenvironment. By targeting metabolic imbalance and oxidative stress, the Metazyme‐containing materials offer a new paradigm for inflammation resolution and fibrosis prevention.

## 4 Materials and Methods

### 4.1 Materials, Cell Lines, and Animals

Manganese acetate, copper(II) nitrate trihydrate, zinc nitrate hexahydrate, cobalt nitrate hexahydrate, and tris(4,7‐diphenyl‐1,10‐phenanthroline)ruthenium(II) chloride ([Ru(dpp)_3_]Cl_2_) were purchased from Macklin Biochemical Co., Ltd. (China). Gelatin (type A, derived from porcine skin) was purchased from Sigma‐Aldrich (USA). L‐Lactic acid (LA) was purchased from TCI Development Co., Ltd. (Japan). Potassium hexacyanocobaltate(III) (K_3_[Co(CN)_6_]), methacrylic anhydride (MA), hydroxylamine hydrochloride, glutathione, 5,5’‐dithiobis‐(2‐nitrobenzoic acid) (DTNB), and tetramethylbenzidine (TMB) were all purchased from Aladdin Co., Ltd. (China). Citric acid, natural catalase (CAT), and natural lactate oxidase (LOX) was purchased from Shanghai Rhawn Chemical Technology Co., Ltd. (China). Hydrogen peroxide (H_2_O_2_), glucose, and sodium alginate (SA) were obtained from Sinopharm Chemical Reagent Co., Ltd. (China). Sodium periodate was purchased from Adamas‐beta, Titan Scientific Co., Ltd. (China). The Inhibition and Produce Superoxide Anion Assay Kit and the Lactate Assay Kit were purchased from Nanjing Jiancheng Bioengineering Institute (China). Phosphate‐buffered saline (PBS, 0.01 M, pH 7.2–7.4), Micro Hexokinase (HK) Assay Kit, simulated body fluid (SBF), and Pyruvate Concentration Assay Kit were purchased from Beijing Solarbio Technology Co., Ltd. (China). Bovine serum albumin (BSA) powder and lipopolysaccharide (LPS) were purchased from Biosharp and Labgic Technology Co., Ltd. (China), respectively. Cytokine enzyme‐linked immunosorbent assay (ELISA) kits and all antibodies were purchased from Boster Biological Technology Co., Ltd. (China). Glucose Detection Kit (O‐toluidine method), 2’,7’‐dichlorodihydrofluorescein diacetate (DCFH‐DA) reactive oxygen species (ROS) Assay Kit, Hoechst 33342 Staining Solution (100×), and BCA Protein Assay Kit were purchased from Beyotime Biotechnology (China). ELISA kits for Pyruvate Dehydrogenase Kinase 1 (PDK1) and M2‐Pyruvate Kinase (PKM2) were purchased from Jiangsu Meimian Industrial Co., Ltd. (China). Ultrapure water was prepared using a Millipore Milli‐Q system. High‐glucose Dulbecco's Modified Eagle Medium (DMEM), fetal bovine serum (FBS), penicillin‐streptomycin, and a spectra multicolor broad range protein ladder were purchased from Thermo Fisher Scientific Inc. (USA).

NCTC clone 929 (L929, RRID: CVCL_0462) cells were purchased from the Cell Bank of the Chinese Academy of Sciences (Shanghai, China). All cell lines were tested and confirmed to be contamination free. Healthy Sprague‐Dawley rats (purchased from Vital River Laboratory Animal Technology Co., Ltd., China) were used for the animal experiments. All procedures involving animals were approved by the Animal Ethics Committee of Dr. Can Biotechnology (Zhejiang) Co., Ltd. (Approval No. DRK‐20250221010).

### 4.2 Synthesis Methods of MnPBA and Metazyme

A total of 0.5 mmol of citric acid and 0.6 mmol of K_3_[Co(CN)_6_] were dissolved in 25 mL of deionized water and sonicated for 10 min to obtain a clear solution A. Separately, 1.1 mmol of manganese acetate was dissolved in 25 mL of water to prepare solution B. Under magnetic stirring at 500 rpm, solution B was added dropwise to solution A, resulting in the immediate formation of a turbid mixture. The suspension was stirred for another 10 min and then aged at room temperature for 24 h. The resulted precipitate was collected by centrifugation, washed three times with water to remove residual reactants, and dried in a vacuum oven at 60°C to yield a white powder, designated as MnPBA (Mn_3_[Co(CN)_6_]_2_). Similarly, ZnPBA, CuPBA, and CoPBA were synthesized by replacing manganese acetate with an equimolar amount of zinc nitrate, copper nitrate, and cobalt nitrate, respectively, following similar procedures.

The as‐prepared MnPBA powder was evenly spread in a ceramic boat, which was then placed in a tube furnace (OTF‐1500X, Hefei Kejing, China) for pyrolysis. After purging the furnace chamber with nitrogen multiple times to remove residual air, the sample was heated to 300°C at a ramp rate of 5°C min^−1^, followed by further heating to 550°C at 2°C min^−1^ under a nitrogen atmosphere. Upon reaching the target temperature, the gas was switched to ammonia (flow rate: 100 mL min^−1^) and maintained for 3 h for thermal treatment. After the reaction, the furnace was purged with nitrogen and allowed to cool naturally to room temperature. To achieve controlled surface passivation, one end of the quartz tube was sealed, and the other end was covered with perforated plastic film to enable gradual exposure to air. The final product was denoted as Metazyme. The same procedures were applied to the ZnPBA, CuPBA, and CoPBA to obtain the ZnN, CuN, and CoN, respectively, for comparison.

### 4.3 Characterization Methods of MnPBA and Metazyme

The morphology of the samples was characterized by field‐emission scanning electron microscopy (SEM; SU8600, Hitachi, Japan) at an accelerating voltage of 4.00 kV. The chemical structures were analyzed using Raman spectroscopy (inVia‐Reflex, Renishaw plc, UK). The crystalline structures were identified by powder X‐ray diffraction (XRD; D8 Advance, Bruker, Germany), with powder samples densely packed on a glass substrate (depth: 0.5 mm), scanned from 10.0° to 90.0° at a rate of 5.0° min^−1^. The diffraction patterns were compared with standard PDF cards (MnO#PDF07‐0230, MnO_2_#PDF43‐1455, Co_4_N#PDF41‐0943) for phase identification.

To further investigate the microstructures, transmission electron microscopy (TEM; JEM 2100, JEOL, Japan), spherical aberration‐corrected scanning TEM (AC‐STEM; Themis Z, Thermo Fisher Scientific, USA), and X‐ray photoelectron spectroscopy (XPS, K‐Alpha, Thermo Fisher Scientific, USA) were employed. Synchrotron‐based X‐ray absorption fine structure (XAFS) measurements were conducted at the Shanghai Synchrotron Radiation Facility, focusing on Co‐K and Mn‐K edges. Data analysis was performed using Athena and Artemis software. The fitting of Co‐K edge data was carried out in k‐space (1.0–10.0 Å^−1^) and R‐space (1.0–3.5 Å), based on paths derived from the Co_4_N crystal structure. The fitting model included single‐scattering paths of Co–N (first shell), Co–Co (second shell), and Co–N–Co bridges (third shell). Key fitting parameters included amplitude reduction factor (S_0_^2^ = 0.78, determined from standard fitting), coordination number (N), bond length (r), Debye‐Waller factor (σ^2^), and energy shift (Δ*E*_0_). To reduce fitting variables, σ^2^ was initially fixed at 0.003, which is consistent with reported Debye–Waller factors for Co–Co and Co–N paths in cobalt nitride and related transition‐metal nitride systems [32, 52]. Upon iterative refinement, the Co–N coordination number converged to ∼2, consistent with the Co_4_N structure, and σ^2^ was refined to ∼0.01. Therefore, the final fitting was performed with N(Co–N) fixed at 2. Multiple fittings were conducted to ensure convergence and physical relevance.

### 4.4 Catalytic Abilities of Metazyme

#### 4.4.1 LOX‐Like Activity of Metazyme

To evaluate the lactate oxidase (LOX)‐like activity of artificial enzymes with different metal centers, 5 mM L‐lactate was incubated with 100 µg mL^−1^ of ZnN, MnN (Metazyme), CuN, or CoN artificial enzymes in 1 mL of phosphate buffer (pH 7.2–7.4) at room temperature for 30 min. After centrifugation, the remaining lactate concentration in the supernatant was determined using a commercial lactate assay kit (Nanjing Jiancheng Bioengineering Institute, China). Absorbance was measured with a microplate reader (Infinite M200 Pro, Tecan, Switzerland). In addition, time‐dependent generation of pyruvate from 5 mM lactate by Metazyme (100 µg mL^−1^) was monitored under the same conditions. At predetermined time points, the supernatant was collected after centrifugation, and the pyruvate concentration was quantified using a pyruvate assay kit (Solarbio, Beijing, China) according to the manufacturer's instructions.

To systematically investigate the enzyme‐mimicking kinetics of Metazyme, 100 µg mL^−1^ of Metazymes was incubated with L‐lactate of a series of concentrations (0, 1, 2, 6, 12, 24, 40, and 80 mM) in phosphate buffer (pH 7.2–7.4) for 10 min at room temperature. After centrifugation, the lactate concentration in the supernatant was measured. To test the dosage dependence, L‐lactate at a series of concentrations was also incubated with Metazyme at a reduced dosage of 50 µg mL^−1^ for comparison. The initial reaction rate (*V*) at each substrate concentration was calculated and fitted using the Michaelis‐Menten equation (Formula 1) to obtain the Michaelis constant (*K_M_*) and the maximum reaction rate (*V_max_*) [53, 54]. The data were also linearized using the Lineweaver‐Burk equation (Formula 2) for validation [53]:

(1) $$V=\frac{\mathrm{conc}.\times V_{\max}}{\mathrm{conc}.+K_{M}}$$

(2) $$\frac{1}{V}=\frac{K_{M}}{V_{max}}\frac{1}{conc.}+\frac{1}{V_{max}}$$

The apparent turnover number (*k*_*cat*,*app*_) of the artificial enzyme was calculated by normalizing the maximum reaction rate (*V_max_*) to the total molar concentration of metal species in the reaction system (Formula 3). The metal contents were determined by inductively coupled plasma mass spectrometry (ICP‐MS, Agilent 7900, Germany), in which Co and Mn species both contribute as potential catalytic centers. The catalytic efficiency (*k*_*cat*,*app*_/*K_M_*) of the Metazyme was also calculated.

(3) $$k_{cat,app}=\frac{V_{max}}{{\left[Metal\right]}_{total}}$$

The catalytic activity of Metazyme and natural LOX under different pH conditions was evaluated by incubating 6 mM L‐lactate with 100 µg mL^−1^ of catalyst (Metazyme or LOX) in phosphate buffer under 25°C at pH 5.0, 7.0, and 9.0, respectively. The catalytic activity of Metazyme and natural LOX under different temperatures was also evaluated at 25°C, 37°C, and 60°C with fixed pH of 5.0, respectively. After a 30 min reaction followed by centrifugation, the lactate concentration in the supernatant was quantified using a lactate assay kit.

#### 4.4.2 CAT‐Like Activity of Metazyme

The kinetic studies were performed by measuring the initial reaction rates (*V*) at various H_2_O_2_ concentrations (0.5, 1, 2, 5, 10, and 20 mM) using Metazyme at dosages of 100 and 50 µg mL^−1^. The data were fitted to the Michaelis‐Menten equation (Formula 1) and Lineweaver‐Burk equation (Formula 2) to determine the Michaelis constant (*K_M_*) and the maximum reaction rate (*V_max_*). The apparent turnover number (*k*_*cat*, *app*_) and catalytic efficiency (*k*_*cat*,*app*_/*K_M_*) of CAT‐like activity were also determined, with Co and Mn species contributing as potential catalytic centers. To quantify H_2_O_2_ concentrations, titanium(IV) sulfate [Ti(SO_4_)_2_] was employed as a colorimetric probe. Specifically, 50 µL of reaction supernatant was mixed with 150 µL of [Ti(SO_4_)_2_] solution (100 mM, in 2 M H_2_SO_4_) in a 96‐well plate. After incubation, the absorbance at 405 nm was measured using a microplate reader. A standard calibration curve was established for quantification (Figure S24).

The catalytic activity of Metazyme and natural CAT under different pH conditions was also evaluated by incubating 5 mM H_2_O_2_ with 100 µg mL^−1^ of catalyst (Metazyme or CAT) in phosphate buffer under 25°C at pH 5.0, 7.0, and 9.0, respectively. The catalytic activity of Metazyme and natural CAT under different temperatures was also evaluated at 25°C, 37°C, and 60°C with fixed pH of 9.0, respectively. After a 30 min incubation, followed by centrifugation, the H_2_O_2_ concentration in the supernatant was quantified using [Ti(SO_4_)_2_] assay.

To systematically evaluate the CAT‐like activity of Metazyme, the decomposition of 1 mM H_2_O_2_ in phosphate buffer (pH 7.2–7.4) was monitored in real‐time using a dissolved oxygen meter (InLab OptiOx, Mettler Toledo, Switzerland). The dissolved oxygen concentration was continuously recorded over 0–10 min using a dissolved oxygen electrode, enabling quantitative assessment of the oxygen generation capacity of the catalyst.

#### 4.4.3 Catalytic Stability and Selectivity

To evaluate the stability of LOX‐like and CAT‐like catalytic activities of Metazyme, a ten‐cycle catalytic test was performed to assess its reusability under specific conditions with the Metazyme concentration of 100 µg mL^−1^. Each cycle was carried out using either 1 mM lactate or 1 mM H_2_O_2_ as the substrate, with a reaction time of 20 min. After each catalytic reaction, the Metazyme was collected by centrifugation, washed three times with PBS, and reused in the next cycle to test its catalytic activity. In addition, the storage stability of Metazyme was examined by incubating the material in PBS for 7 days. The catalytic activities of Metazyme after PBS incubation were then evaluated under identical conditions using 100 µg mL^−1^ Metazyme with 5 mM L‐lactate or 5 mM H_2_O_2_ for 30 min, and were compared with those of freshly prepared Metazyme. The catalytic stability of Metazyme in different physiological media was assessed by dispersing the material in PBS, simulated body fluid (SBF), and SBF supplemented with 1 mg mL^−1^ BSA. After incubation under the same conditions, the LOX‐like and CAT‐like catalytic activities were measured to evaluate the influence of buffer composition and protein‐rich environments on the catalytic performance.

To investigate the substrate selectivity of Metazyme, its catalytic performance was evaluated against several common substrates using the following protocols. The **SOD‐like activity** was determined using the Inhibition and Generation of Superoxide Anion Radical Determination Kit. The Metazyme was used at a concentration of 100 µg mL^−1^. Supernatants were collected at 0, 5, 10, and 20 min, respectively, and absorbance was measured at 550 nm according to the manufacturer's instructions. In the presence of a superoxide radical scavenger, the absorbance of the test sample is expected to be lower than that of the control. The **OXD‐like activity** was measured based on a previously reported method [55]. Briefly, 100 µg mL^−1^ Metazyme was mixed with 0.5 mM TMB in 1 mL of PBS (pH 7.2–7.4). Supernatants were collected at 0, 5, 10, and 20 min, respectively, and absorbance was recorded at 650 nm. The **POD‐like activity** was also assessed following literature protocols [55]. In this assay, 100 µg mL^−1^ Metazyme was mixed with 100 mM H_2_O_2_ and 0.5 mM TMB in 1 mL of PBS (pH 7.2–7.4). Supernatants were collected at the same time points (0, 5, 10, and 20 min), and absorbance was measured at 650 nm. The **glucose oxidase (GOx)‐like activity** was evaluated using the Glucose Detection Kit (O‐toluidine method). A reaction mixture containing 100 µg mL^−1^ Metazyme and 5 mM glucose in 1 mL PBS (pH 7.2–7.4) was incubated, and supernatants were collected at 0, 30, 60, and 90 min, respectively. The glucose concentrations were measured according to the kit instructions and quantified using a standard curve (Figure S25). The **glutathione peroxidase (GPx)‐like activity** was determined using the DTNB colorimetric method from the literature [56]. A mixture of 100 µg mL^−1^ Metazyme and 5 mM glutathione in 1 mL PBS (pH 7.2–7.4) was incubated, and supernatants were collected at 0, 30, 60, and 90 min, respectively. For each sample, 50 µL of the supernatant was added to 0.15 mL of 0.15 mol L^−1^ NaOH, followed by 50 µL of 3% formaldehyde. After reacting for 2 min at pH 8.0 and 25°C, the mixture was further reacted with 0.2 mL DTNB solution for 5 min at 25°C. Absorbance was measured at 412 nm for comparison.

### 4.5 Preparation of Rgel and MetaRgel

Rgel is a composite hydrogel synthesized by polymerization of methacrylated gelatin (GelMA) and oxidized sodium alginate (ox‐SA). MetaRgel is a functionalized hydrogel obtained by incorporating Metazymes into the Rgel precursor solution before crosslinking. The detailed synthesis procedures were described in the Supporting Information. To prepare the microgel precursors, 0.25 wt% LAP photoinitiator solution (solution L) was prepared in PBS and used to dissolve 10 wt% GelMA and 4 wt% ox‐SA, respectively. The Rgel precursor solution was obtained by mixing 5 mL of GelMA solution, 2.5 mL of ox‐SA solution, and 2.5 mL of solution L, followed by ultrasonication for degassing. For MetaRgel, 0.8 mL of Metazyme dispersion (5 mg mL^−1^ in solution L) was additionally added to the above mixture, and the volume of solution L was adjusted to 1.7 mL to maintain a constant total volume and concentration of initiators. The rod‐shaped microgels were fabricated using a microfluidic‐assisted UV polymerization approach. The prepolymer solution served as the aqueous phase, and paraffin oil containing 15 v% Span80 acted as the oil phase. Droplets were formed in a microchannel with an inner diameter of 250 µm at flow rates of 500 µL h^−1^ (aqueous) and 1000 µL h^−1^ (oil). Upon formation, the droplets were immediately exposed to 320 W UV light (INTELLIRAY 400, Uvitron, USA) for crosslinking in situ. To prevent premature gelation of GelMA, the aqueous phase syringe was wrapped with a heating coil. The morphology of the microgels was captured under an optical microscope (IX81, Olympus, Japan). The cured microgels were sequentially washed three times with hexane, ethanol, and deionized water to remove residual surfactant and unreacted chemicals, and stored in PBS at 4°C until equilibrium swelling was achieved. The stability of the MetaRgel and the retention behavior of Metazyme were further evaluated in PBS under physiological conditions. At predetermined time points over a 3‐day period, the supernatant was collected and passed through a centrifugal filter with a pore size of 5 µm to remove intact microgels. The resulting filtrate was analyzed by flow cytometry (FACScalibur, Becton, Dickinson and Company, USA) to detect free Metazyme particles. A Metazyme suspension prepared in PBS was used as a positive control to define the particle gating strategy, while PBS alone served as a negative control.

### 4.6 Cell Culture, Biocompatibility Evaluation, and Inflammation‐Hypoxia Regulatory Effects

L929 fibroblasts were cultured in high‐glucose DMEM supplemented with 10% FBS, 100 U mL^−1^ penicillin, and 100 µg mL^−1^ streptomycin under standard conditions (37°C, 5% CO_2_). The cytotoxicity was assessed using the CCK‐8 assay. L929 cells were seeded into Transwell plates (24‐well plate, 0.4 µm polycarbonate membrane) at a density of 1 × 10^4^ cells per well (400 µL medium per well) and cultured for 12 h. The upper chamber was loaded with different test samples: 50 mg mL^−1^ of Rgel or MetaRgel microgels, or free Metazymes (at a final concentration of 0.02 mg mL^−1^, equivalent to that in the MetaRgel group). After 24 h of incubation, the cells were gently washed with PBS and each medium was replaced with 400 µL of fresh medium containing 40 µL of CCK‐8 reagent. Following a 2 h incubation at 37°C, 100 µL of each medium was transferred to a 96‐well plate, and the absorbance at 450 nm was measured using a microplate reader. The cell viability was calculated according to Formula 4:

(4) $$\mathrm{Cell}\;\mathrm{viability}\;\left(\%\right)=\frac{OD_{sample}-\bar{OD_{negative}}}{\bar{OD_{positive}}-\bar{OD_{negative}}}\times 100\%$$

where $\bar{OD_{negative}}$ and $\bar{OD_{positive}}$ represent the mean absorbance of the negative (medium + CCK‐8 only) and positive control groups (cells without materials), respectively, and *OD_sample_* is the absorbance of the treated samples.

To evaluate the regulatory effects of the materials on intracellular ROS and hypoxic conditions, a Transwell co‐culture system (24‐well plate, 0.4 µm polycarbonate membrane) was used. L929 cells were seeded in the lower chamber at 5 × 10^4^ cells per well in 1 mL of culture medium, and allowed to adhere for 12 h. The medium was then replaced with 0.8 mL of fresh complete medium, and the cells were subjected to one of three stimuli: hypoxia (1% O_2_), lipopolysaccharide (LPS, 400 ng mL^−1^), or lactate (20 mM). Simultaneously, the upper chamber was loaded with different test samples: 50 mg mL^−1^ of Rgel or MetaRgel microgels, or free Metazyme (at a final concentration of 0.02 mg mL^−1^, equivalent to that in the MetaRgel group).

After 24 h of co‐culture, the cells in the lower chamber were washed three times with PBS and stained for fluorescence imaging: (1) ROS detection was performed using a staining solution containing 10 µM DCFH‐DA and 5 µM Hoechst 33342 (for nuclear staining); (2) hypoxia detection was conducted using 10 µg mL^−1^ [Ru(dpp)_3_]Cl_2_ and 5 µM Hoechst 33342. The cells were incubated in the dark at 37°C for 20 min, followed by PBS washing. Fluorescence images were acquired using a fluorescence microscope (Olympus IX81, Japan). Semi‐quantitative analysis was performed by ImageJ to measure the mean intensity value and the positive area. The total fluorescence intensity was obtained by multiplying these two parameters.

### 4.7 Evaluation of Reprogramming of Metabolism and Fibrosis‐Related Cytokines In Vitro

6‐Well Transwell plates (0.4 µm polycarbonate membrane) was employed to establish a pathological model in vitro for the systematic evaluation of the effects of Rgel, MetaRgel, and Metazyme on glucose metabolism and inflammation in L929 cells under hypoxia (1% O_2_), LPS (400 ng mL^−1^), and lactate (20 mM) stress conditions. The L929 cells were seeded in the lower chamber at a density of 4 × 10^5^ cells per well in 2.5 mL of complete culture medium. After 12 h of incubation at 37°C with 5% CO_2_, the medium was replaced with fresh medium containing the pathological stimuli, and the upper chamber was supplemented with one of the following treatments: Rgel or MetaRgel (final concentration 50 mg mL^−1^), or free Metazymes (final concentration 0.02 mg mL^−1^).

After 24 h of co‐culture, the supernatants were collected for quantitative analysis of extracellular lactate using a commercial lactate assay kit, and for pro‐inflammatory cytokines IL‐6 and TGF‐β1 using mouse ELISA kits, following the instructions of manufacturer.

For intracellular glycolytic analysis, the cells were harvested via trypsinization and washed with PBS. HK activity was determined and normalized to cell number following the manufacturer's instructions. Protein expression of PDK1 and PKM2 was analyzed following cell lysis in RIPA buffer supplemented with protease inhibitors. The total protein concentration was quantified using the BCA assay for normalization. All protein extraction and enzymatic activity assays were performed on ice to preserve protein function.

### 4.8 Rat Full‐Thickness Wound Model In Vivo

A dorsal full‐thickness splinted wound model was established in Sprague‐Dawley rats to assess the wound‐healing efficacy of different treatments. Healthy female rats (200–250 g, n = 15 per group) were randomly assigned to three groups: Rgel‐treated, MetaRgel‐treated, and saline control. Each group was further divided into three time points (days 3, 6, and 12), with five rats sacrificed and analyzed at each time point for biological assessments. For surgical procedures, the rats were anesthetized via intraperitoneal injection of 3 wt% pentobarbital sodium solution (0.25 mL per rat). After shaving and sterilizing the dorsal skin, two circular full‐thickness wounds (8 mm in diameter, spacing > 15 mm) were created using a sterile biopsy punch. To simulate the mechanical tension of human skin wounds, sterile splints were sutured around each wound using 6–8 interrupted stitches to maintain wound openness and prevent wound contraction.

The wounds were treated topically with Rgel or MetaRgel dressings, while the control group received saline treatment only. All wounds were covered with 3 M breathable dressings, which were replaced every 3 d along with the fresh treatment application. The wound healing progression was monitored by standardized digital photography, and the wound area was quantitatively analyzed using ImageJ software. The wound closure percentage was calculated according to Formula 5. For wound‐size analysis, values from the two wounds were averaged within each rat, and statistical comparisons were performed at the animal level.

(5) $$Woundclosure\left(\%\right)=\left(1-\frac{Woundarea}{Initialwoundarea}\right)\times 100\%$$

To evaluate the biosafety of the treatments in vivo, blood samples were collected from rats on postoperative day 6 for serum biochemical analysis, including alanine aminotransferase (ALT), aspartate aminotransferase (AST), blood urea nitrogen (BUN), creatinine (CR), and alkaline phosphatase (ALP). At the experimental endpoint, the rats were euthanized via intraperitoneal injection of an overdose of pentobarbital sodium. For histological evaluation, the wound tissues were harvested on postoperative day 12 and fixed in 4% paraformaldehyde. Hematoxylin and eosin (H&E) staining and Masson's trichrome staining were performed to comprehensively assess epidermal regeneration, inflammatory infiltration, and collagen deposition. In addition, the major organs (heart, liver, spleen, lung, and kidney) were harvested and subjected to H&E staining for biosafety evaluation in vivo. All stained sections were scanned using a digital slide scanner (Pannoramic MIDI, 3DHISTECH, Hungary). For each rat, multiple fields from two wound sites were quantified using ImageJ software and averaged, yielding one biological replicate per rat.

For molecular‐level analysis, the wound tissues were collected on days 3 and 6 post‐surgery, snap‐frozen in liquid nitrogen, and analyzed using ELISA to quantify key cytokines involved in the wound microenvironment. 100 mg of tissue from each rat was homogenized using an automated tissue grinder (JXFSTPRP‐24L, Jingxin, China) in 1 mL of PBS containing protease inhibitors. The homogenates were centrifuged at 10 000 rpm for 15 min at 4°C, and the supernatants were collected for subsequent analysis. The total protein concentration was determined using the BCA assay for data normalization. High‐sensitivity ELISA kits were used to quantify key cytokines in the wound microenvironment, including TNF‐α, TGF‐β1, and IL‐6. In addition, the lactate levels in the wound tissues were quantified using a commercial lactate assay kit following the manufacturer’s protocol, and the results were normalized to the total protein content.

In addition, immunohistochemical (IHC) staining was performed on wound sections collected on days 6 and 12 to evaluate the spatial expression and abundance of glycolysis‐ and fibrosis‐related proteins. All IHC‐stained sections were scanned using a digital slide scanner, and ImageJ software was used to quantify the positively stained areas. Each time point included 5 biological replicates to ensure statistical robustness.

### 4.9 Statistical Analysis

All experiments were conducted independently at least three times, as indicated in the corresponding figure captions. Data are presented as mean ± standard deviation (SD). Statistical analysis was performed using GraphPad Prism. One‐way analysis of variance (ANOVA) was used for comparisons among three or more groups, and two‐way ANOVA was applied where multiple factors were involved. For two‐group comparisons, unpaired t‐tests were used. Statistical significance was denoted as follows: *p < 0.05, **p < 0.01, ***p < 0.001, and ****p < 0.0001.

## Conflicts of Interest

The authors declare no conflict of interest.

## Supporting information




**Supporting File**: advs74705‐sup‐0001‐SuppMat.docx.

## Data Availability

The data that support the findings of this study are available from the corresponding author upon reasonable request.;
