# Supplementary material for: Bifunctional Artificial Enzymes‐Loaded Microgels With LOX‐ and CAT‐Like Activities for Metabolic Reprogramming and Scarless Wound Repair
Source: Adv Sci (Weinh). 2026 Mar 6;13(27):e21248. doi: 10.1002/advs.202521248 (PMC13170211; doi:10.1002/advs.202521248)
Supplement: Supplementary file 1 — Supporting File: advs74705‐sup‐0001‐SuppMat.docx. [file ADVS-13-e21248-s001.docx]

**Supporting Information**

**Bifunctional Artificial Enzymes-Loaded Microgels with LOX- and CAT-Like Activities for Metabolic Reprogramming and Scarless Wound Repair**

*Yongyuan Kang, Pai Peng, Liang Song**,* *Qiaoxuan Wang, Zilong Zhong, Dongchao Qiu, Yunfan Liu, Kefei Zhao, Xiaofei Dong, Changyou Gao^*^*

Yongyuan Kang, Pai Peng, Liang Song, Qiaoxuan Wang, Zilong Zhong, Dongchao Qiu, Yunfan Liu, Kefei Zhao, Xiaofei Dong, Changyou Gao

Zhejiang Key Laboratory of Advanced Organic Materials and Technologies, MOE Key Laboratory of Macromolecular Synthesis and Functionalization, Department of Polymer Science and Engineering, Zhejiang University, No. 866 Yuhangtang Road, Hangzhou 310058, China

Xiaofei Dong, Changyou Gao

Zhejiang Engineering Research Center for Interface Technology of Medical Polymers and Devices, Shaoxing Key Laboratory of Healthcare Materials and Application Technology, and Center for Healthcare Materials, Shaoxing Institute, Zhejiang University, Shaoxing 312099, China

Dr. Li Dak Sum & Yip Yio Chin Center for Stem Cell and Regenerative Medicine, Zhejiang University, Hangzhou 310058, China

Corresponding author: E-mail: cygao@zju.edu.cn (C. Gao)

## Synthesis of GelMA and ox-SA

GelMA were synthesized according to a previously reported protocol.^[1]^ Briefly, 4 g of gelatin was dissolved in 50  mL of water at 40  °C. The pH was adjusted to 7-8 using 1 M NaOH, followed by the slow addition of 400 μL of methacrylic anhydride (MA) pre-diluted in 5  mL of DMF. After the reaction mixture was stirred at 40 °C for 12 h, the product was purified via dialysis against water for 7 d and lyophilized to obtain the solid GelMA. The chemical structure of GelMA was characterized by ^1^H nuclear magnetic resonance spectroscopy (NMR; DMX 400 MHz, Bruker, Germany), as shown in Figure S12. Oxidized sodium alginate (ox-SA) was synthesized following a literature method.^[2,3]^ Specifically, 10  g of sodium alginate was dispersed in 50  mL of ethanol, and 15 mmol of sodium periodate dissolved in 50 mL of water was added dropwise under stirring. After reacting for 6 h, the resulted solid was collected by centrifugation, redissolved in water, dialyzed for 7 d, and freeze-dried to yield the final ox-SA powder. The chemical structure of ox-SA was characterized by ^1^H NMR (Figure S13). The degree of oxidation was quantified using a hydroxylamine hydrochloride titration method.^[4]^ Specifically, 50 mg of ox-SA was dissolved in 20 mL of 0.25 M hydroxylamine hydrochloride solution, and whose pH value was adjusted to 4.50 using 1 M NaOH. The solution was stirred at room temperature for 24 h, followed by titration with 0.1 M NaOH until the pH was recovered to 4.50, with continuous monitoring using a pH meter (PHS-3C, INESA Scientific Instrument Co., Ltd., China). The volume of NaOH consumed was recorded and used to calculate the degree of oxidation. Each sample was titrated in triplicate, and the average value was reported. The degree of oxidation calculated using Formula S1 was 12.8%.

$$\begin{aligned} Degree of oxidation (\%)=\frac{198\times V\left( NaOH \right)\times conc.\left( NaOH \right)}{2\times w\left( oxSA \right)}\times100\%\#\left( S1 \right) \end{aligned}$$

## Characterization of Rgel and MetaRgel

To assess swelling behavior, the swollen weight ($W_{w}$) of the microgels in PBS or deionized water was compared with their dry weight ($W_{d}$) after lyophilization (Figure S15). The swelling ratio was calculated according to Formula S2:

$$\begin{aligned} Swelling ratio \left( \% \right)=\frac{W_{w}-W_{d}}{W_{d}}\times100\%\#\left( S2 \right) \end{aligned}$$

Mechanical properties were evaluated using cylindrical microgel scaffolds collected by vacuum filtration. Oscillatory rheological measurements were performed at 25 °C using a rheometer (RS6000, HAAKE, Germany) equipped with a 20 mm parallel plate geometry. Frequency sweep tests were conducted at a fixed strain of 5% over the range of 0.1-100 Hz to determine the storage modulus (G’) and loss modulus (G”) (Figure S17). Self-healing performance was assessed using a step-strain test under a constant frequency of 1 Hz by alternating the strain between 5% and 500% (Figure S18).

## References

[1] Z. Chen, X. Hu, Z. Lin, H. Mao, Z. Qiu, K. Xiang, T. Ke, L. Li, L. Lu, L. Xiao, "Layered GelMA/PEGDA Hydrogel Microneedle Patch as an Intradermal Delivery System for Hypertrophic Scar Treatment," ACS Applied Materials & Interfaces (2023): 43309–43320, https://doi.org/10.1021/acsami.3c06800.

[2] S. Reakasame, A. R. Boccaccini, "Oxidized alginate-based hydrogels for tissue engineering applications: A review," Biomacromolecules (2018): 3–21, https://doi.org/10.1021/acs.biomac.7b01331.

[3] H. Liao, H. Zhang, W. Chen, "Differential physical, rheological, and biological properties of rapid in situ gelable hydrogels composed of oxidized alginate and gelatin derived from marine or porcine sources," Journal of Materials Science: Materials in Medicine (2009): 1263–1271, https://doi.org/10.1007/s10856-009-3694-4.

[4] A. Jejurikar, X. T. Seow, G. Lawrie, D. Martin, A. Jayakrishnan, L. Grøndahl, "Degradable alginate hydrogels crosslinked by the macromolecular crosslinker alginate dialdehyde," Journal of Materials Chemistry (2012): 9751–9758, https://doi.org/10.1039/C2JM30564J.

[5] J. Yu, Y. He, Y. Wang, S. Li, S. Tian, "Ethylenediamine-oxidized sodium alginate hydrogel cross-linked graphene oxide nanofiltration membrane with self-healing property for efficient dye separation," Journal of Membrane Science (2023): 121366, https://doi.org/10.1016/j.memsci.2023.121366.

Figure S1. Raman spectrum of Metazyme

Figure S2. XRD patterns of MnPBA precursor and Metazyme.

Figure S3. XRD pattern of Metazyme nanozyme with standard reference peaks.
The diffraction pattern of Metazyme (black) is compared with standard PDF cards for MnO (PDF#07-0230, green), MnO_2_ (PDF#43-1455, red), Co_4_N (PDF#41-0943, blue), and graphite (PDF#41-1487, gray).

Figure S4. Lactate consumption by nanozymes containing different central metal ions (ZnN, MnN (Metazyme), CuN, and CoN). Data are presented as mean ± SD (n = 3). *P < 0.05, ***P < 0.001 (one-way ANOVA).

Figure S5. Generation of pyruvate by oxidation of lactate (5 mM) under catalyzation of Metazyme (100 μg mL^-1^) along with time prolongation. Data are presented as mean ± SD (n = 3).

Figure S6. Lineweaver-Burk plots showing the LOX-like catalytic kinetics of Metazyme measured at 50 μg mL^-1^. Data are presented as mean ± SD (n = 3).

Figure S7. Lineweaver-Burk plots showing the CAT-like catalytic kinetics of Metazyme measured at 50 μg mL^-1^. Data are presented as mean ± SD (n = 3)


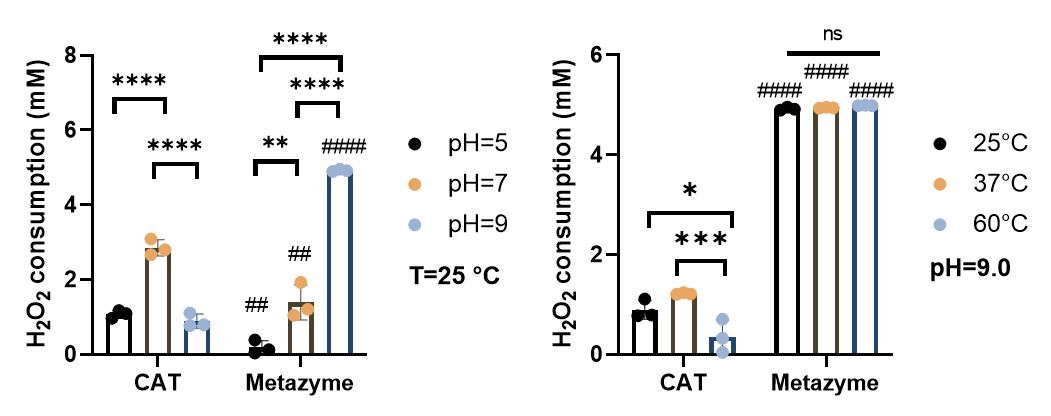


Figure S8. Comparison of H_2_O_2_ consumption by natural CAT and Metazyme under different pH and temperature conditions. n = 3. ns = not significant. *P < 0.05, **P < 0.01, ***P < 0.001, and ****P < 0.0001 (one-way ANOVA between selected groups). ##P < 0.01 and ####P < 0.0001 (t-test between natural enzymes and Metazyme under the same conditions).


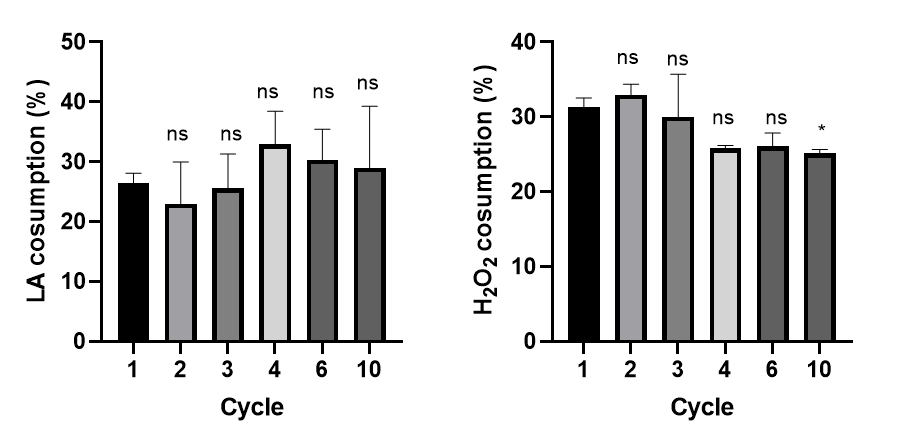


Figure S9. Cyclic catalytic performance of Metazyme for (left) lactate oxidation (LOX-like activity) and (right) H_2_O_2_ decomposition (CAT-like activity). Data are presented as mean ± SD (n = 3). ns = not significant, *P < 0.05 (one-way ANOVA compared with cycle 1).


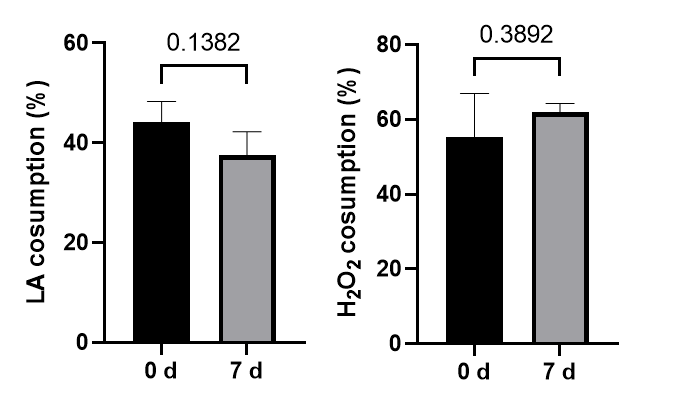


Figure S10. LOX-like (left) and CAT-like (right) catalytic activities of fresh Metazyme and Metazyme incubated in PBS for 7 days. Data are presented as mean ± SD (n = 3). P values are marked insets (t-test).


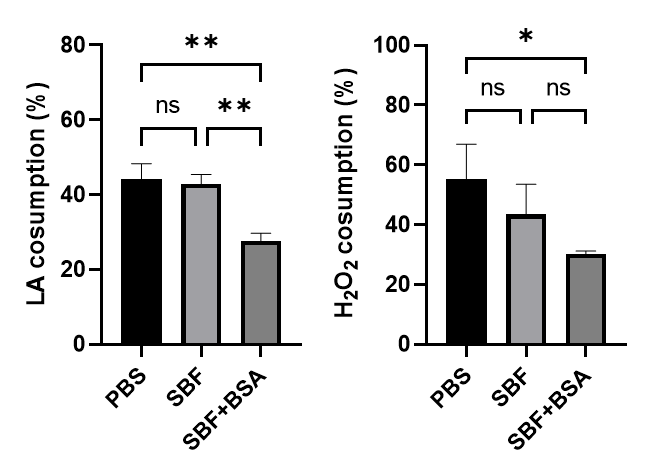


Figure S11. LOX-like (left) and CAT-like (right) catalytic activities of fresh Metazyme and Metazyme being incubated in PBS for 7 days. Data are presented as mean ± SD (n = 3). ns = not significant, *P < 0.05, and **P < 0.01 (one-way ANOVA).

Figure S12. ^1^H NMR spectrum of methacrylated gelatin (GelMA) in D_2_O.


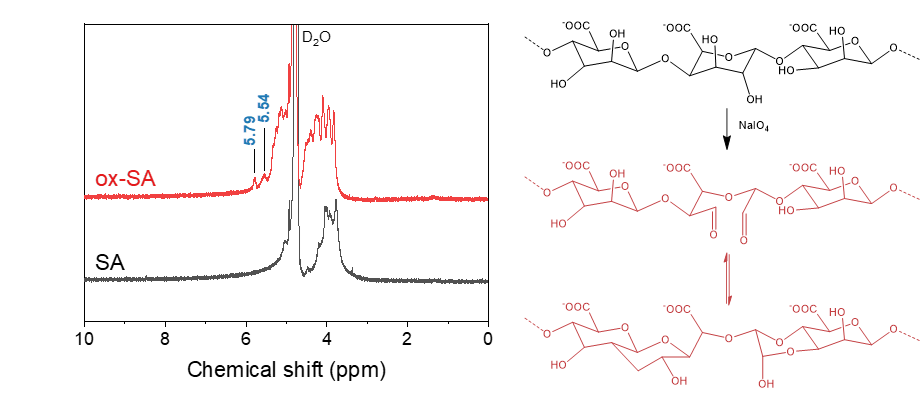


Figure S13. ^1^H NMR spectra of sodium alginate (SA, black) and oxidized sodium alginate (ox-SA, red) in D_2_O.

The appearance of new peaks at 5.79 and 5.54 ppm in ox-SA corresponds to hemiacetal protons formed by the reaction between aldehyde groups and adjacent hydroxyl groups, indicating successful oxidation.^[2,5]^


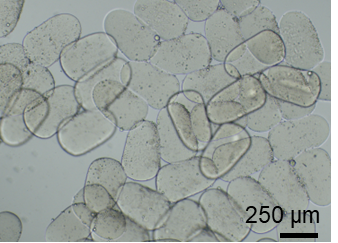


Figure S14. Optical microscopy image of rod-shaped microgels fabricated via microfluidic emulsification.


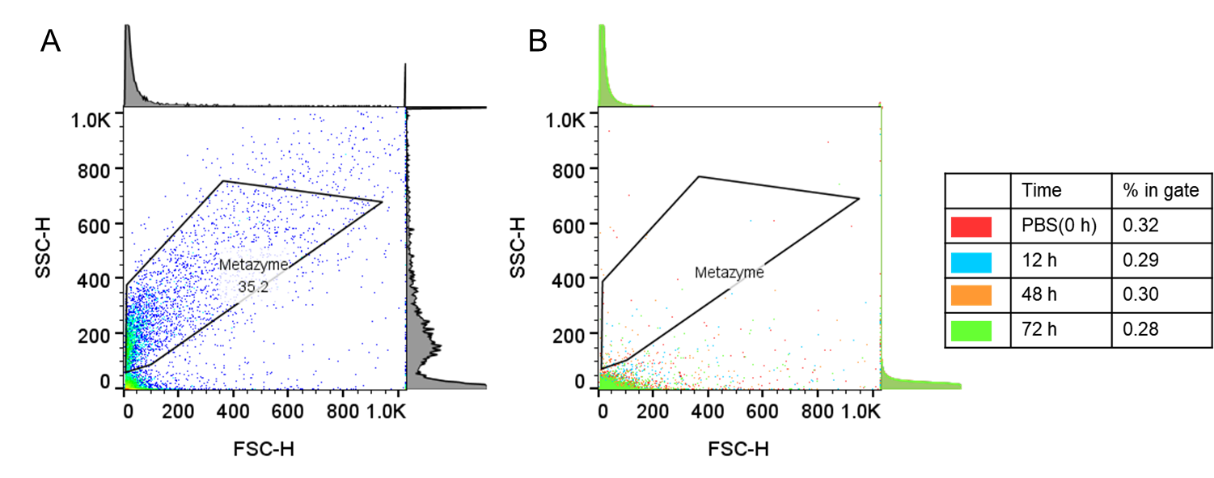


Figure S15. Flow cytometry gating strategy for detecting free Metazyme in PBS after incubation with MetaRgel.

Figure S16. Swelling behavior of Rgel and MetaRgel in deionized water and PBS at room temperature. Data are presented as mean ± SD (n = 3).

The MetaRgel showed a higher swelling ratio than Rgel in water, possibly owing to that the dark-colored Metazymes absorbed light and quenched radicals during photopolymerization, while their surface groups interfered with Schiff-base crosslinking, together reducing network density. Both microgels exhibited reduced swelling in PBS due to ionic crosslinking effects.


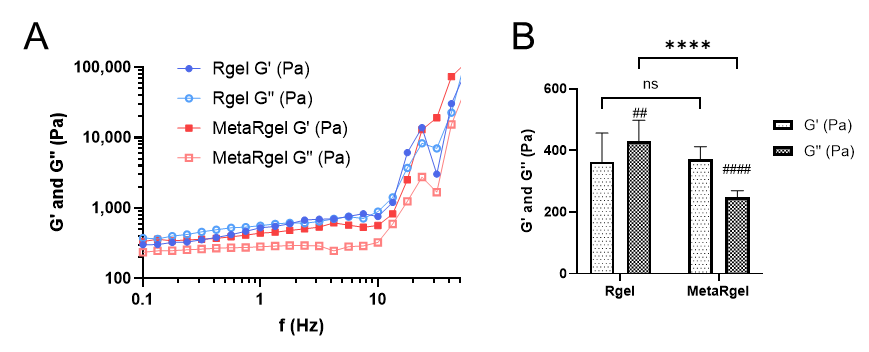


Figure S17. Rheological characterization of Rgel and MetaRgel microgels. (A) Storage modulus (G’) and loss modulus (G”) measured by oscillatory shear rheometer using a parallel plate geometry over a frequency sweep. (B) Quantification of average moduli showing the viscoelastic properties of each group. Data are presented as mean ± SD (n = 3). ns=not significant, ****P < 0.0001 (t-tests between selected groups). ##P < 0.01 and ####P < 0.0001 (t-tests between G’ and G” within the same group).


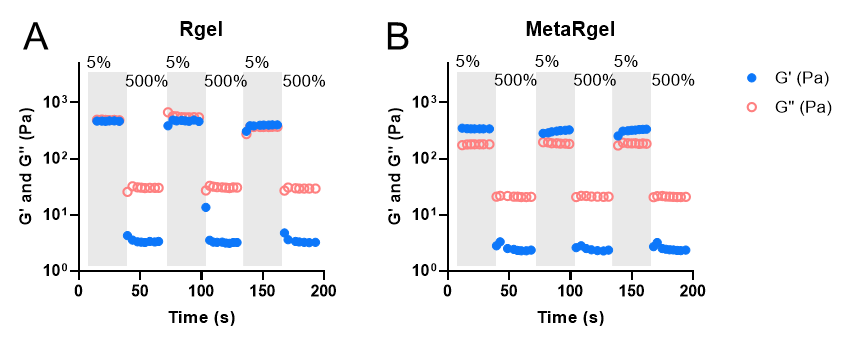


Figure S18. Strain-dependent rheological behavior of Rgel and MetaRgel microgels. Storage modulus (G’) and loss modulus (G”) of Rgel (A) and MetaRgel (B) under different oscillatory strain.

Both the Rgel and MetaRgel exhibited decreased moduli at high strain, indicating enhanced flowability that facilitates syringe extrusion. Notably, under low-strain conditions, the MetaRgel exhibited G’ values that were clearly higher than G”, indicating predominant solid-like behavior to maintain structural integrity after injection.

Figure S19. Viability of L929 cells after treatment with Rgel, Metazyme, and MetaRgel compared to control. Data are presented as mean ± SD (n = 5). *P < 0.05, ***P < 0.001 (one-way ANOVA).


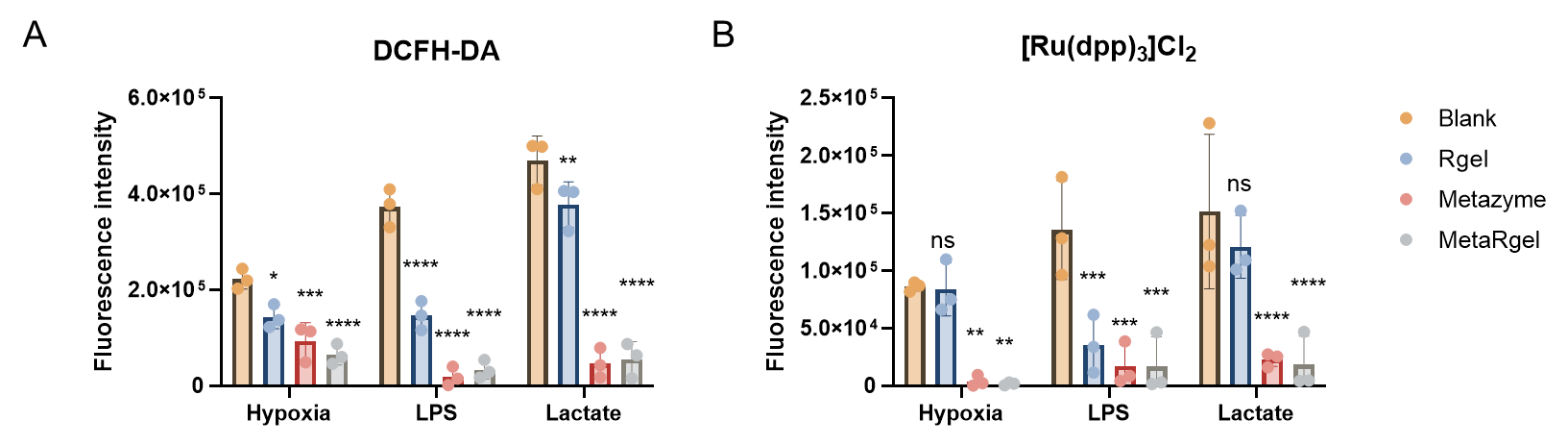


Figure S20. Semi-quantitative fluorescence intensity analysis of intracellular ROS and hypoxia in L929 cells after different treatments. Data are presented as mean ± SD (n = 3). *P < 0.05, **P < 0.01, ***P < 0.001, and ****P < 0.0001. ns, not significant compared to the Blank (two-way ANOVA followed by Dunnett’s multiple comparisons, comparing each treatment to the Blank within the same condition).


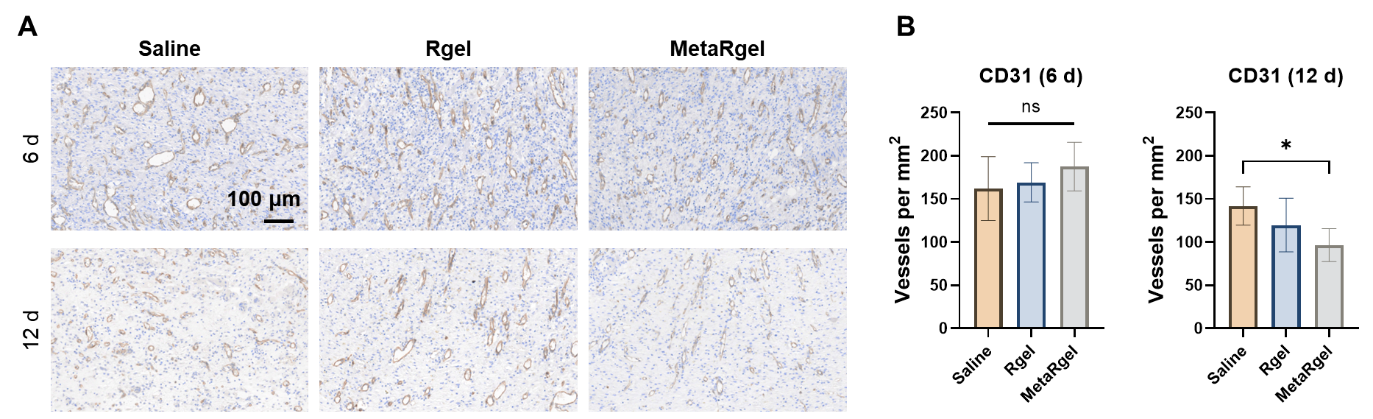


Figure S21. (A) Representative IHC staining images of CD 31 (day 6 and 12). (B) Quantification of CD31-positive vessels. Data are presented as mean ± SD. n = 5 biologically independent samples. *P < 0.05. ns, not significant (one-way ANOVA).


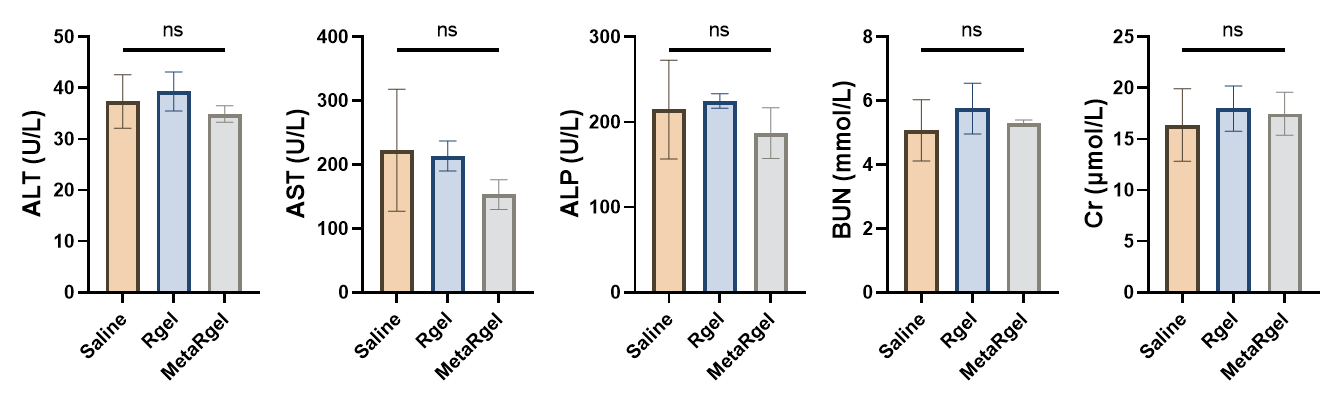


Figure S22. Serum levels of alanine aminotransferase (ALT), aspartate aminotransferase (AST), alkaline phosphatase (ALP), blood urea nitrogen (BUN), and creatinine (Cr) measured on day 6 post-treatment. Data are presented as mean ± SD. n = 5. ns, not significant (one-way ANOVA).


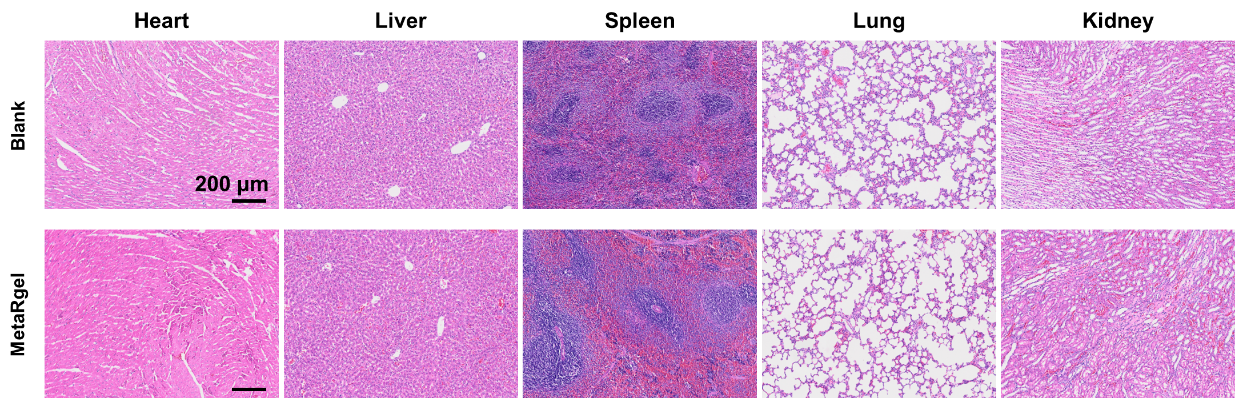


Figure S23. H&E-stained sections of the heart, liver, spleen, lung, and kidney from Blank and MetaRgel-treated rats.

Figure S24. Standard curve of H_2_O_2_ concentration.

Figure S25. Standard curve of glucose concentration.
